# Converging chemistry and clinical orthopedics in the emerging role of MOFs in advanced bone defect repair

**DOI:** 10.7150/thno.129987

**Published:** 2026-05-11

**Authors:** Yangmengfan Chen, Xiaoyang Liu, Xuming Chen, Hao Du, Zongke Zhou

**Affiliations:** Department of Orthopedics and Research Institute of Orthopedics, West China Hospital, Sichuan University, Chengdu, 610041, China.

**Keywords:** metal-organic frameworks, bone regeneration, drug delivery, ion therapy, composite scaffolds, osteogenesis, angiogenesis

## Abstract

In recent years, metal-organic frameworks (MOFs) have attracted significant attention in regenerative medicine due to their exceptional structural tunability, high surface-to-volume ratios, and controllable porosity. This review systematically outlines the versatile functions of MOFs in bone defect repair, including their use as sustained ion release systems for osteogenic metal ions (*e.g.*, Sr^2+^, Zn^2+^, Mg^2+^, or Cu^2+^), nanocarriers for controlled delivery of biomolecules (*e.g.*, growth factors, drugs, or genes), and functional components within composite scaffolds to enhance mechanical and biological properties. Moreover, MOFs exhibit inherent antibacterial and anti-inflammatory properties, which are also important for bone defect repair. We critically discuss current challenges, including biostability, degradation kinetics, and long-term biosafety, and highlight perspectives on future directions, including the design and development of the smart, stimuli-responsive MOF systems for bone defect repair.

## 1. Introduction

### 1.1. Clinical Challenges in Bone Defect Repair

Bone not only provides mechanical support and protects our internal organs, but also stores massive amounts of essential minerals. Nevertheless, the prevalence of bone diseases, including osteoarthritis, bone fractures, bone cancer, and osteomyelitis, continues to rise globally. In China alone, more than 6 million orthopedic cases are reported annually. Similarly, in the United States, the number of orthopedic cases is projected to increase by 30% from 2005 to 2025, while in Europe, the number of cases is projected to rise by 28% from 2010 to 2025 1.

Currently, several strategies exist for bone defect repair, including the use of metallic implants, autografts, or allografts. However, these approaches suffer from intrinsic limitations 2. First, the corrosion of metallic implants often elicits a foreign body reaction, increasing the risk of aseptic loosening and revision surgery. Second, the use of autografts is limited by donor-site scarcity and susceptibility to postoperative infections. Third, allografts may increase risks of pathogen transmission and adverse immunogenic responses, thereby restricting their therapeutic application 3. Moreover, several concerns include insufficient biocompatibility, inadequate mechanical stiffness, and poor osseointegration 4.

A successful bone defect repair involves a well-orchestrated biological process comprising three overlapping and distinct activities: inflammation, new bone formation, and bone remodeling 5. The initial inflammatory response is critical for clearing debris and initiating the subsequent regenerative programs. Importantly, the inflammation must be resolved in time to prevent the occurrence of chronic inflammation 6. Then, the new bone formation is initiated by the formation of the soft callus, and subsequently through neovascularization, and osteogenesis of bone marrow mesenchymal stem cells (BMSCs) 7. Finally, in the bone remodeling phase, both osteoblasts and osteoclasts are coordinated to remodel the micro-structure, and enhance bone strength 8.

To promote the repair of bone defects, bone tissue engineering has reshaped the field by focusing on creating bioactive constructs that actively support regeneration. Recent tissue regeneration and engineering strategies rely on the synergistic combination of three key components: biomaterials that simulate the structure of native extracellular matrix (ECM), and are loaded with bioactive components that guide cellular behavior. Among the various nanomaterials explored, metal-organic frameworks (MOFs) are a particularly promising platform for bone defect repair due to their hybrid architecture, exceptional structural tunability, high porosity, and ability to incorporate bioactive components. More importantly, MOFs can be manufactured to align with the specific demands during bone defect repair: (1) modulating inflammation 9, (2) enabling controlled delivery of osteogenic and angiogenic factors during the repair phase 10, and (3) sustained release of bioactive ions to facilitate bone remodeling 11. Therefore, this multifunctionality of MOFs confers the ability to dynamically interact with the bone repair microenvironment 12, which not only compensates for the drawbacks of conventional treatments for bone defect repair, but also promotes the development of bone tissue engineering.

### 1.2. Evolution of Bone Tissue Engineering

Bone tissue engineering strategies can accelerate bone defect repair by creating functional and biological substitutes 13. Orthopedic implants in the early years were generally passive and bioinert, providing mechanical support. However, the conventional implant often fails to integrate with host bone tissue, leading to undesirable outcomes 14. This recognition prompted the transition from passive to bioactive orthopedic implants, marking a key evolution in bone tissue engineering.

Advanced bone tissue engineering strategies mainly rely on 3 fundamental principles: (1) using 3D scaffolds to simulate ECM and offer mechanical stiffness 15, (2) loading stem cells 16, and (3) delivering bioactive factors to promote cell proliferation, differentiation, and matrix formation 17. In this way, bone tissue engineering evolved beyond merely passive mechanical roles and actively directed bone defect repair through spatiotemporal release of bioactive molecules 18, providing cell-adhesive ligands 19 and topographical cues 14. In the development of advanced bioengineering, nanomaterial-based approaches, such as MOFs, offer distinct promise. Many biochemical methods rely on administering high concentrations of growth factors or cytokines to directly influence cellular activities, but are often limited by short *in vivo* half-lives, off-target effects, and high costs 20. In contrast, MOFs offer tailorable catalytic activities 21, efficient loading capacity, spatiotemporally controlled release 22, and even provide physical cues to guide cell fate by modifying nano-/micro-topography 23. Both the unique physiochemical property and programmable bioactivity transform MOFs from passive carriers into a dynamic platform of actively and synergistically promoting bone defect repair 24.

### 1.3. MOFs as Versatile Biomaterials

**Figure 2:**
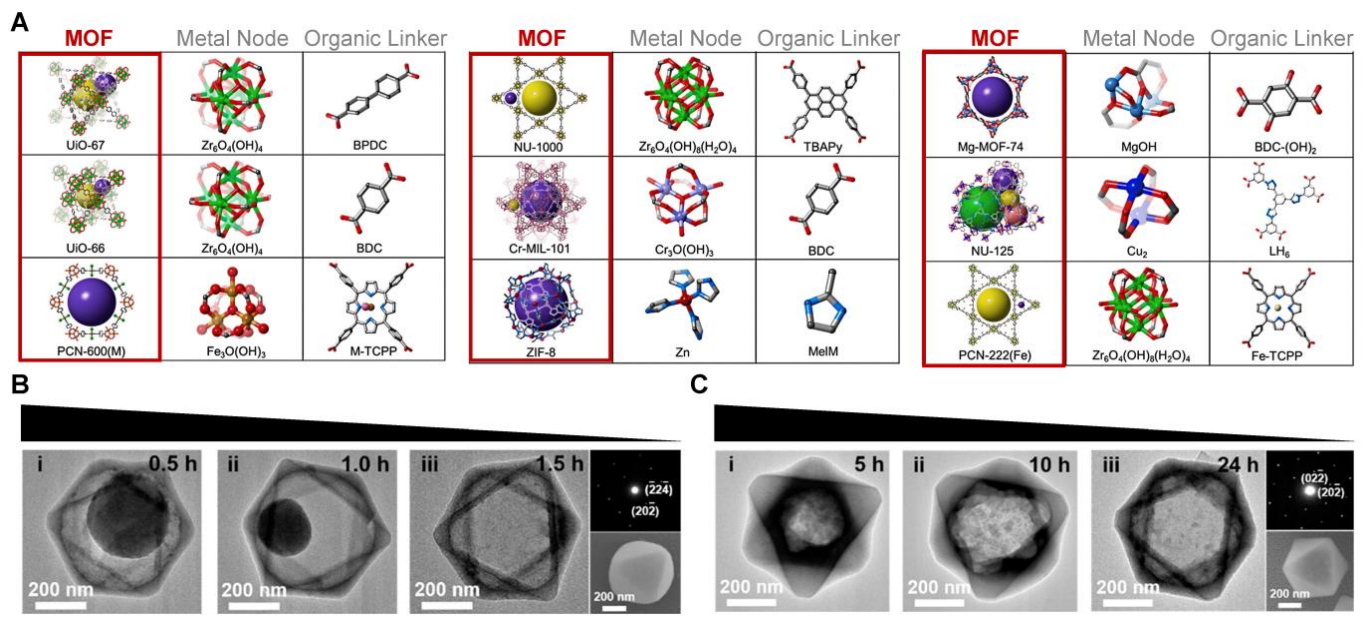
**Structural diversity and sequential degradation profiles of MOFs. (A)** Representative crystal structures and their corresponding metal nodes/organic linkers of selected MOFs (UiO-67, UiO-66, PCN-600 (M), NU-100, Cr-MIL-101, ZIF-8, Mg-MOF-74, NU-125, PCN-222(Fe)). **(B)** Transmission electron microscopy (TEM) images of UiO-66-(OH)_2_@UiO-66-Br core-shell nanoparticle in a ROS-enriched environment after 0.5, 1.0, and 1.5 h, demonstrating rapid structural disintegration. **(C)** Sequential TEM images of the same UiO-66-(OH)_2_@UiO-66-Br nanoparticle in an HNO_3_-enriched environment after 5, 10, and 24 h, showing a comparatively slower degradation profile. Adapted with permission from 36, copyright 2016 American Chemical Society, 37 copyright 2019 American Chemical Society.

Since the first report by Yaghi *et al.* in 1995, MOFs have rapidly evolved in recent decades 25. Their multifunctionality and tunable properties in biological environments have attracted growing interest in biomedical research, particularly for theranostic applications. Structurally, MOFs are presented as crystalline porous biomaterials that consist of specific metal ions and multidentate organic linkers 26. The hybrid architecture offers exceptional structural diversity and functionality, such as ultrahigh surface areas, precisely customizable porous structures, and adjustable pore sizes 27. These features are important for targeted delivery and controlled release, and also crucial for biomedical applications in bone tissue engineering 28. Their porosity, ranging from microporous to mesoporous, facilitates efficient encapsulation of diverse functional cargos through van der Waals forces, π–π interactions, and H-bonding, and protects them from the undesirable degradation, thereby improving their *in vivo* stability and functionality 29. The function of MOFs can be modulated through (1) selection of metal nodes and functionalized organic linkers, (2) control of crystal size and morphology, (3) construction of MOF-based composites and hybrids, and (4) synthesis of MOF-derived biomaterials via different treatments 30. Accordingly, MOFs can generally be classified into major families, such as, isoreticular MOFs (IRMOFs), zeolitic imidazolate frameworks (ZIFs), porous coordination networks (PCNs), Materials of Institute Lavoisier (MIL), and others **(Figure 2A)**. Although structurally distinct, covalent organic frameworks (COFs) are often mentioned alongside MOFs because of their analogous porosity 31.

In recent years, many advanced synthetic strategies, including multivariate synthesis, post-synthetic modification, and topology-guided design, have been increasingly developed and employed. These approaches enable atomic-level engineering of MOF architectures, further optimizing their functionality for complex biomedical applications 32

### 1.4. Connecting MOF Nanoarchitecture to Bone Healing Processes

MOFs are increasingly recognized as promising biomaterials due to their unique structure and functions 33. Specifically, MOFs’ ability to load and release diverse osteoinductive factors (*e.g.*, growth factors, bioactive ions, and drugs) either through sustained diffusion or in response to specific stimuli 34, allows precise control of the local therapeutic microenvironment. This capability avoids the limitations of systemic therapies, which often face issues like poor biocompatibility, off-target effects, and dose-limiting toxicity in conditions like osteoporosis and bone cancer 35.

This review highlights MOFs as dynamic platforms capable of orchestrating bone defect repair. We analyze how the rational design of MOF nanoarchitectures, including therapeutic ion reservoirs, controlled release of bioactive molecules, and composite scaffolds, enables multifaceted regulation of the regenerative microenvironment promoting osteogenesis, angiogenesis, modulating immune responses, and controlling infection. This review provides a comprehensive framework for designing next-generation MOF-based systems to overcome current challenges in bone tissue engineering and to facilitate the translation of laboratory innovations into clinical applications.

## 2. Fundamental Aspects of MOF Design for Bone Regeneration

MOFs are made from metal nodes and organic linkers. These materials can be customized and degrade under certain conditions **(Figure 2B-C)**. This makes MOFs very useful for helping bone regeneration 38. Their effectiveness depends on some key design factors that work synergistically. First, selecting the right metal nodes and organic linkers directly determines MOF properties, such as osteogenic, anti-osteoclastic, and antimicrobial activities, as well asdegradation behavior and drug-loading capacity. Second, changing their porosity and morphology at the nano- and micro-scale helps cell infiltration, nutrient exchange, and acts like natural bone. Third, improving the biocompatibility and biosafety of MOFs by using safe components and checking the host tissue response. These points are important for designing better MOFs to improve bone defect repair.

### 2.1. Synthesis and Applications of MOFs

The healing abilities of MOFs in bone repair come from their atomic and molecular structure. Traditional biomaterials have fixed properties, but MOFs show a unique "synthesis-structure-function" paradigm. The choice of production process directly affects their physicochemical functions, degradation kinetics, and clinical applications 39. Understanding how different synthetic strategies for MOF manufacturing can convert them into functional biomaterials is crucial.

#### 2.1.1. Conventional Synthesis Methods

These established methods produce MOFs with well-defined crystal structures, which primarily affect their stability and porosity.

1) Solvothermal/Hydrothermal Method: This method makes MOF crystals using a solution with metal salts and organic linkers at high pressure and heat (usually 80-200 °C). It helps dissolve reactants and promotes high-quality crystal growth, yielding highly crystalline and porous MOFs. For bone repair, it is a good choice for making stable MOFs like Zr-based UiO-66, which act as strong, long-lasting reservoirs, allowing sustained ion release (*e.g.*, Sr^4+^ from Sr-MOFs) over weeks or months to support bone healing 40.

2) Microwave-Assisted Synthesis: Microwave irradiation speeds up nucleation, cutting down production time from days to hours or even minutes 41. This method tends to produce smaller, more uniform nanoparticles, which can directly affect cell uptake efficiency, bio-distribution, and degradation rate. MOFs produced this way usually exhibit nanoscale dimensions, which enable them to enter target cells and affect cellular processes 42.

3) Ultrasound (US)-Assisted Synthesis: This method uses sound waves to cause the formation of microbubbles that quickly collapse, forming hot spots. This leads to the fast formation of MOFs under relatively mild conditions 43. This method is especially useful for making MOF-based drug delivery systems for bone repair, because it forms very organized structures at low temperatures, thereby protecting thermo-sensitive biomolecules 44. The MOFs made this way usually have uniform particle sizes and remain stable in liquid, which helps their biomedical use 45. For example, an MWCNT/Fe_3_O_4_/Cu(BDC) nanocomposite synthesized with US helped drug loading at 99.6% efficiency and kept a sustained release kinetics, showing strong bacteria-killing abilities 46. Also, Cu-MOF made this way exhibit appears as round nanoparticles (size: 65 nm, with 10 nm pores) and can inhibit bacteria proliferation as low as 100 ppm 47. In summary, US-assisted synthesis offers an efficient, controlled method to make MOFs with optimal physicochemical properties for bone repair.

#### 2.1.2. Advanced and Precision Synthesis Methods

With the development of interdisciplinary technologies, many methods enable precise control over the shape, surface, and specific functions of MOFs.

1) Modulated Synthesis: Organic linkers for metal coordination sites, such as acetic acid 48 or benzoic acid 49, can precisely tune crystal size, shape, and surface chemistry. These changes directly affect protein adsorption and cell-MOF interactions. Thus, the MOF itself can guide stem cell differentiation or regulate immune responses without needing other biochemical signals.

2) Post-Synthetic Modification (PSM): PSM means changing and manufacturing of MOFs that were already made through the use of methods like covalent grafting, coordination exchange, or guest encapsulation. This approach allows MOFs to exhibit specific biological functions without changing their basic structure. For example, ketoprofen was added to Mg-MOF-74 using PSM, thereby preserving the crystal form of Ket@Mg-MOF-74 while enabling sustained drug release and synergistic Mg^2+^-mediated osteogenic and anti-inflammatory effects 50.

3) Mechanochemical Synthesis: Applying mechanical force through grinding or milling causes reactions between metal nodes and linker precursors 51. This technique enables close mixing of MOFs with polymers or bioceramics, further enhancing their mechanical properties and bioactivity 52.

Overall, the synthesis strategy is the first and most critical design choice that affects a MOF's properties and biological effects, including long-lasting release, targeted delivery, and catalytic activity. Because synthesis strategies can confer these fundamental properties to MOFs, they are important for the development of advanced MOF-based biomaterials in the context of bone repair.

### 2.2. Composition and Functional Design

The metal ions in MOFs are not inactive; they function as therapeutic agents. For instance, Zn^2+^ 53 and Mg^2+^ 50 are important for initiating osteogenesis, while Sr^2+^ can dualistically enhance new bone formation and suppress osteoclast function 54. Similarly, Cu^2+^ can stabilize hypoxia-inducible factor-1α (HIF-1α), thereby helping the formation of new blood vessels, which are critical for bone repair 55. The organic component of MOFs also helps regulate their biological activity. Recent studies have used bioactive molecules, such as amino acids, peptides, or endogenous metabolites, instead of conventional nitrogen- or oxygen-centered ligands. As a result, this innovative strategy converts MOFs from passive frameworks to active biomaterials 56.

This high level of customizability allows improvement in many physical properties of MOFs. These include surface topography, mechanical stress distribution, and surface charge. These features play an important role in the transduction of signaling molecules tand shaping the microenvironment 57. These factors are key for attracting bone cells, guiding their behavior, and helping minerals form at the implant-bone interface 58. Moreover, if MOFs are designed to match the composition and elastic modulus of native bone, they can distribute weight more naturally and avoid bone loss caused by the stress-shielding effect. For instance, Matlinska *et al.* created bio-MOFs using Ca^2+^ and Sr^2+^ with a bisphosphonate linker, providing therapeutic ions and anti-osteoporotic molecules, which helped protein adsorption and bone cell proliferation 59. Similarly, Wang *et al.* added Mg-MOF-74 and silk fibroin to a 3D-printed titanium (Ti) implant, creating a coating that effectively reduced stiffness mismatch, alleviated stress shielding, and resulted in significantly improved bone growth and osseointegration 60. These studies underscore the paradigm of tailoring MOF composition to achieve specific mechano-biological outcomes in bone repair.

### 2.3. Biomimetic Nano-/Micro-Structural Engineering for Bone Matrix Recapitulation

Bone has a hierarchically organized structure; the cortical bone is dense with layers, while the cancellous bone has a porous and network-like structure. This graded structure of bone, with changing porosity and smooth shifts in stiffness and flexibility, is important for its biomechanical function 61. This structural hierarchy also affects cell behavior by providing biophysical cues. In bone tissue engineering, scaffold architecture, especially nano- and microtopographical features, serves as a critical source of these cues. These features control cell phenotype, adhesion, viability, and overall therapeutic efficiency 62.

MOF-based scaffolds provide a specialized platform for simulating the complex structure of bone through precise control of the nano- and micro-architecture. By adjusting parameters such as pore size distribution, surface roughness, and pore interconnectivity, MOFs can be designed to imitate natural bone. This biomimetic approach can provide structural activity and modulate certain biological responses 63. For instance, nanoscale surface topography can regulate the local immune environment by by driving macrophage polarization toward a pro-regenerative phenotype 64. Microscale porosity and interconnectivity play an important role in forming new vasculature and delivering nutrients, creating a microenvironment suitable for bone regeneration 65.

### 2.4. Biocompatibility and Biosafety

The translational potential of MOFs for bone repair depends on their inherent biosafety. A major issue is MOF degradation in the body, which may lead to burst release of metal ions and organic ligands. This release might cause cytotoxicity, inflammatory responses, or systemic toxicity 66. Also, as exogenous nanoparticles, MOFs are susceptible to immune recognition, macrophage phagocytosis, and unintended inflammatory activation, which can compromise their delivery efficiency and therapeutic outcome 67.

Several strategic approaches have been developed to mitigate these risks. First, choosing safe components is the key, using natural or endogenous metal ions *(e.g.*, Zn^2+^, Mg^2+^, Ca^2+^) and organic linkers from the body or approved by the U.S. Food and Drug Administration (FDA). Second, manufacturing surfaces through coating with polymers like hyaluronic acid or polyethylene glycol (PEG), or by using biomimetic cell membranes, can improve stability, reduce immune responses, and improve safety 67. An example is Pt@ZIF-8@La, which combines biocompatible Zn^2+^, FDA-approved lanthanum, and platinum nanozymes, showing a successful strategy to combine therapeutic function with *in vitro* and *in vivo* biosafety 68. Therefore, when designing MOFs for bone repair, critical issues, including safety and stability, must be carefully evaluated, is essential for maximizing the therapeutic efficacy of MOF-based implants.

## 3. MOFs Function as Therapeutic Ion Reservoirs

Metal ions are important in biological systems, regulating functions such as signal transduction, bone formation, and enzymatic activity. The controlled release of specific metal ions is a smart way to modulate them 69. *In vivo*, metal ions are released when upon MOF separation. Several metal ions like Zn^2+^, Mg^2+^, Sr^2+^, Fe^3+^, and Ti^2+^ attracted widespread attention for their ability to promote new bone formation by advancing osteogenesis. Many osteogenic MOFs made with these metal ions showed that sustained ion release is important for supporting the mineral deposition 70.

### 3.1. Magnesium-Based MOFs

Magnesium-based MOFs (Mg-MOFs) show significant potential in bone regeneration by modulating the senescent microenvironment and enhancing osteogenesis. Mg-Ce-MOF scaffolds efficiently eliminate reactive oxygen species (ROS), thus delaying BMSC senescence. Sustained Mg^2+^ release activates the Nrf2 signaling pathway and upregulates ALDH3A1 expression, further counteracting cellular aging. These scaffolds also promote M2 macrophage polarization, generating an osteoimmune microenvironment that promotes osteogenic differentiation and accelerates bone defect repair in aged models 71.

A study introduced a dual-network injectable hydrogel composed of a complex of a Mg^2+^-gallate-based MOF and osteogenic peptide-coated GelMA-ODex 72. Mg^2+^ in this system is very important for its bioactivity. When the MOF breaks down, the released Mg^2+^ enhances migration and tube formation in human umbilical vein endothelial cells (HUVEC), by upregulating VEGF and HIF-1α gene expression. This robust vascularization is crucial for later bone regeneration. Although the main effect on bone growth was mediated by OGP, the Mg^2+^-driven angiogenic response was necessary to create a favorable microenvironment for angiogenesis and osteogenesis. *In vitro* and *in vivo* studies showed that the composite hydrogel effectively scavenged ROS, promoted soft-tissue healing, and significantly improved bone repair. This study underscored Mg^2+^ as a key therapeutic ion in making multifunctional biomaterials for bone defect repair 72.

Choi *et al.* fabricated a novel nano-engineered hydrogel, incorporating Ca- and Mg-based MOFs. Mg^2+^ promoted osteogenic differentiation of pre-osteoblasts by regulating integrin-mediated signaling via activating MAPK signaling, and regulating key enzymes like alkaline phosphatase (ALP). This function complemented Ca^2+^-driven mineralization, creating a synergistic effect that potently increased OPN and *OCN* gene expression and mineral deposition *in vitro*. Controlled release of Mg^2+^ also contributed to immunomodulation and ROS scavenging *in vivo*, preventing excessive inflammation and supporting a conducive microenvironment for healing. The combined and sustained delivery of Mg^2+^ and Ca^2+^ from the hydrogel scaffold significantly accelerated bone defect repair *in vivo*, demonstrating that Mg^2+^ is an important component in this advanced therapeutic platform for bone repair 73.

### 3.2. Copper-Based MOFs

**Figure 3:**
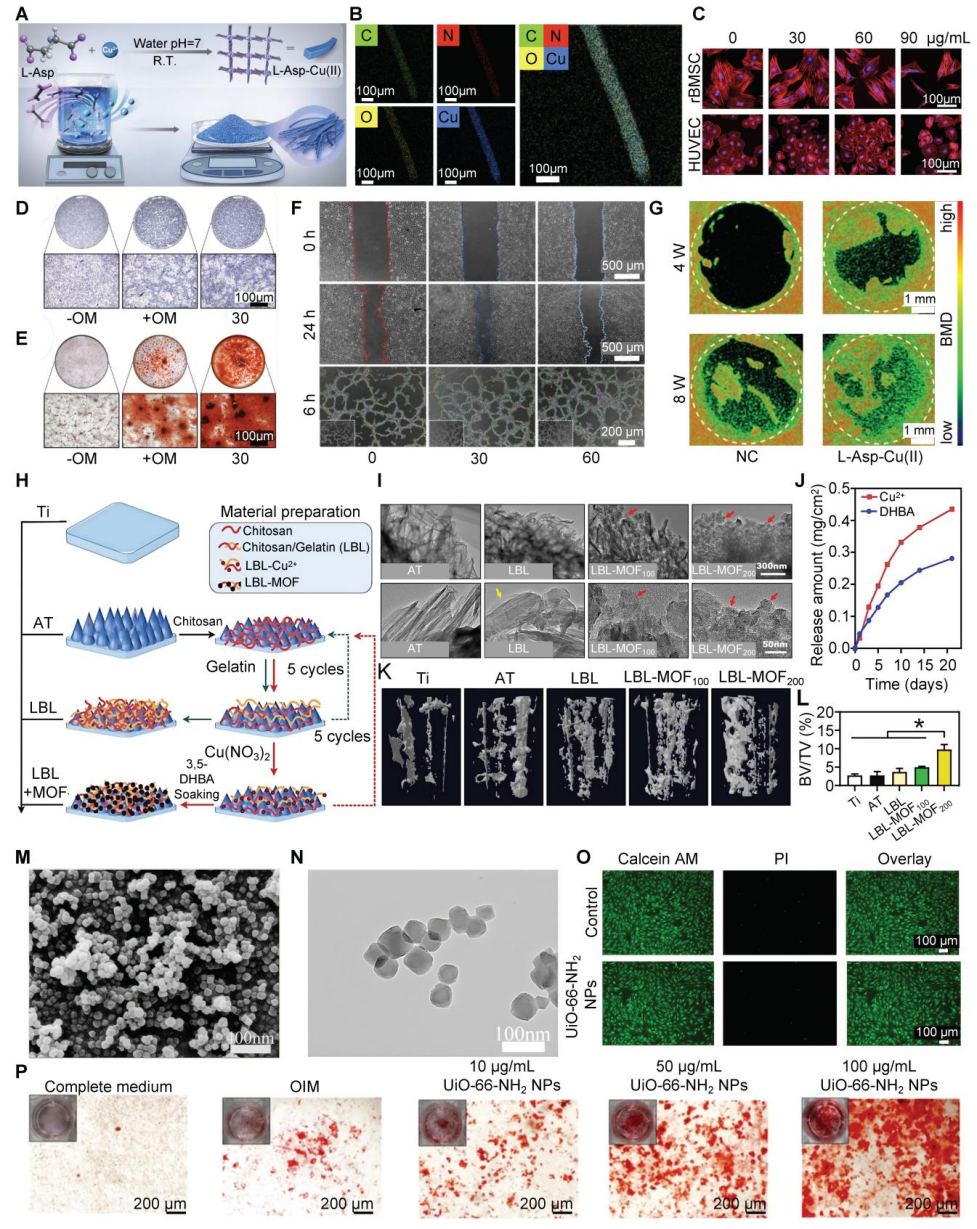
** Synthesis, characterization, and multimodal bioevaluation of L-Asp-Cu (II) MOF and its coating for bone defect repair. (A)** Schematic of L-Asp-Cu (II) MOF fabrication. **(B)** Material characterization: elemental mapping. **(C)** Cytocompatibility assessment: fluorescence images of BMSCs/HUVECs (cytoskeleton: red, TRITC-phalloidin; nuclei: blue, DAPI) after culture with MOF (0-90 μg/mL), showing intact morphology. **(D)** Early osteogenesis: ALP staining at day 7. **(E)** Late osteogenesis: ARS staining at day 21. **(F)** Angiogenesis *in vitro*: scratch assay (top) and tube formation (bottom) of HUVECs. **(G)** *In vivo* bone regeneration: micro-CT 3D reconstructions of calvarial defects at 4/8 weeks; defect margin (white dashed circle), bone mineral density gradient shown. **(H)** Coating design: schematic of LBL assembly. **(I)** Coating microstructure: TEM images of AT, LBL, LBL-MOF100, and LBL-MOF200; coating layer (yellow arrow), MOF nanoparticles (red arrows).** (J)** Release kinetics: cumulative release of 3,5-DHBA and Cu^2+^ from LBL-MOF200 over 14 days. **(K)** *In vivo* implant performance: micro-CT images of new bone around implants at 8 weeks. **(L)** Quantitative analysis: bone volume fraction. Adapted with permission from 74, copyright 2025 American Chemical Society; 75, copyright 2025 American Chemical Society.

Copper-based MOFs (Cu-MOFs) help repair bone defects by their sustained release of bioactive Cu^2+^ ions, which aid in new bone and blood vessel formation. One example is L-Asp-Cu(II) bio-MOF, made from L-aspartic acid **(Figure 3A, B)**. This bio-MOF exhibits excellent cytocompatibility **(Figure 3C)** and osteoinductive ability **(Figure 3D, E)** while promoting angiogenesis **(Figure 3F)**. Mechanistically, Cu^2+^ activates the transforming growth factor-β/bone morphogenetic protein (TGF-β/BMP) signaling pathway, upregulating key osteogenic and angiogenic genes, thereby coupling vascularization with bone formation. This synergistic action makes Cu-MOFs promising for treating critical-sized bone defects **(Figure 3G)** 74. Hua *et al.* used a Cu-MOF with a chitosan/gelatin layer-by-layer coating on Ti implants to enhance bone regeneration through neurovascular-bone coupling **(Figure 3H)**. The Cu-MOF, synthesized from Cu^2+^ and neurogenic 3,5- acid **(Figure 3I)**, enabled sustained release of both bioactive components over 21 days** (Figure 3J)**. Released Cu^2+^ strongly promoted *VEGF* expression and angiogenesis. Moreover, the Cu-MOF coating indirectly facilitated osteogenesis by stimulating Schwann cells to secrete neurotrophic factors, thereby enhancing vascularization and osteogenesis. Proteomic analysis revealed activation of PI3K-Akt and TGF-β pathways, underscoring the role of Cu-MOFs in orchestrating multi-tissue regeneration and highlighting their potential as multifunctional bioactive coatings to accelerate implant osseointegration and complex tissue repair **(Figure 3K, L)** 75.

### 3.3. Zinc-Based MOFs

Zinc-based MOFs offer great promise in regeneration medicine because they can support bone growth and protect against oxidative damage. Zn/Co-MOFs scavenge ROS through SOD/CAT-like cascade catalysis, protecting cells from the damage caused by oxidative stress, while also releasing Zn^2+^ that aids new bone formation. Transcriptomic analyses reveal that Zn/Co-MOFs upregulate the Wnt signaling pathway, including key genes like FZD8, FZD9, and GPC4, which are important for osteogenesis. This combination of antioxidant defense with pro-osteogenic ability makes Zn-based MOFs promising for treating tough clinic issue like infection 76.

Li *et al.* create pH-sensitive nanoparticles by using ZIF-8 to encapsulate and deliver minocycline hydrochloride. These nanoparticles were effectively taken up by human periodontal ligament cells (hPDLCs). The released Zn^2+^ reduced inflammatory cytokines in the local area via the AKT/GSK3β/NRF2 pathway, decreasing bone resorption and increasing bone density *in vivo* 77.

### 3.4 Fe-Based MOFs

Fe-based MOFs, particularly those from the MIL, have both high surface area and structural stability 78. For instance, Yu *et al.* synthesized MIL-100 (Fe) via aqueous-phase synthesis, and incorporated Mg into its cages (Mg@MIL-100 (Fe)), then grafted the MOF with polyacrylic acid (PAA). The PAA layer regulated Mg^2+^ release and prevented ion leakage, increasing Mg loading. Released Mg^2+^ promoted osteoblast differentiation and accelerated osteoclast healing. Cytotoxicity assays using the osteoblast-like MG-63 cell line confirmed the biocompatibility of Mg@MIL-100(Fe)-PAA, while the ALP assay demonstrated enhanced osteogenesis 79. Xiong *et al.* explored the synergistic effect of low-intensity pulsed ultrasound (LIPUS) and Fe^3+^ on bone repair. Cell proliferation assays revealed that Fe^3+^ at 400 μg/L exhibited strong pro-osteogenic effects. Moreover, the combination of LIPUS and Fe^3+^ can synergistically enhance osteoblast differentiation, ALP activity, and mineralization by activating Wnt/β-catenin signaling. Thus, this strategy significantly accelerates bone defect repair 80.

Currently, the biocompatibility and drug delivery ability of Fe-MOFs have been recognized; however, the underlying molecular mechanism of Fe involved in bone metabolism requires further in-depth investigations.

### 3.5 Strontium-Based MOFs

Strontium-based MOFs (Sr-MOFs) are promising biomaterials for enhancing bone regeneration, especially in compromised healing environments such as those associated with diabetes. Sr-doped ZIF-8 incorporated into GelMA hydrogels enables sustained, localized release of strontium ions (Sr^2+^), promoting osteoblast activity and inhibiting osteoclast function 54. Sr-MOFs enhance BMSC proliferation and differentiation and upregulate key osteogenic markers, including Runx-2, ALP, OCN, and BMP-2. Additionally, Sr^2+^ modulates the bone immune microenvironment by polarizing macrophages into an M2-like phenotype, thereby inhibiting inflammation and scavenging ROS, creating a favorable regenerative milieu. Therefore, Sr-MOFs represent a multifunctional strategy for bone tissue engineering, combining osteoinductive, immunomodulatory, and anti-oxidative properties to address complex challenges in bone repair 81.

In another study, Wang *et al.* decorated ZnO and Sr(OH)_2_ on the surface of the sulfonated polyetheretherketone (PEEK). The combined Zn^2+^ and Sr^2+^ release not only inhibited the proliferation of bacteria but also promoted osteogenesis in a high-glucose microenvironment. Of note, their work demonstrated that Zn&Sr-SPEEK could restore mitochondrial function by reducing *DLP1* (Dynamin 1-like protein), restoring mitochondrial membrane potential, reducing ROS generation, and significantly improving bone formation 82.

### 3.6. Cobalt-Based MOFs

Cobalt-based alloys are often used as metal implants for bone in medical treatments 83. Cobalt-based MOFs (Co-MOFs) can assist in bone and cartilage regeneration. Qin *et al.* created a bilayer hydrogel using 3D-printing, incorporating ZIF-67 (Co-MOF) in the upper layer and ZIF-8 (Zn-MOF) in the lower layer to repair osteochondral (OC) defects. The Co^2+^ released from ZIF-67 acted as a hypoxia mimetic, stabilizing HIF-1α, thereby activating Wnt/β-catenin signaling, upregulating *SOX9* and *ACAN*, and facilitating hyaline cartilage formation.

*In vivo* studies conducted on rabbits with cartilage defects have shown that a layer containing ZIF-67 greatly enhances cartilage repair. This bilayer design enabled controlled release of Co^2+^ and Zn^2+^, mimicking the natural OC structure and facilitating simultaneous repair of cartilage and subchondral bone simultaneously. This study identified the key biological functions of Co-MOFs in guiding chondrogenesis and their promise for multi-tissue repair 84.

A recent study further showed that Co-doped bimetallic MOFs effectively mitigated inflammation; the catalytic activity of Co scavenged ROS, reducing oxidative stress and inflammation. This strategy activated the Wnt pathway, boosting new alveolar bone formation 85.

### 3.7 Zirconium-Based MOFs

Zirconium (Zr) alloys are widely used in bone implants due to their excellent biocompatibility and strong physical stability 86. Zr-MOFs, such as UiO-66-NH_2_, show potential for bone defect repair because of their biosafety and inherent osteogenic activity. Specifically, Zr ions can enhance the adhesion, proliferation, and osteogenesis of BMSCs. Transcriptomic analyses indicate that UiO-66-NH_2_ upregulates key osteogenic markers and activation of signaling pathways (*e.g.*, PI3K-Akt and MAPK) important for osteogenesis. In addition, its porous nature allows it to load and sustain the release of osteoinductive agents. These features make Zr-MOFs attractive for developing promising biomaterials, as they can support bone healing and mitigate adverse effects 87. When incorporated into bioinks, UiO-66 nanocrystals function as a stable reservoir of Zr ions, ensuring their sustained release and promoting osteogenic differentiation by upregulating osteogenic genes (*e.g.*, BMP2, Runx-2, collagen I (COL-I), OCN, and ALP). This approach significantly enhances the ability of printed scaffolds to promote bone formation, demonstrating that UiO-66 is a highly promising component for advanced bone repair materials 88.

Yan *et al*. prepared a specialized fluorine-containing Zr-MOF film. This film could be very useful for bone implant applications because of its safety and robust osteogenic properties. The film releases fluoride, which can kill bacteria without harming host cells. The added fluorine also helps regulate the release of fumaric acid, which has anti-inflammatory effects. Together, these features make an osteo-friendly microenvironment for bone regeneration and osteointegration 89.

### 3.8 Nickel-Based MOFs

Nickel-Based MOFs (Ni-MOFs) have great potential in bone defect repair, primarily as components of composite scaffolds. As reported by Lin *et al.*, mixing Ni-MOF with β-cyclodextrin via electrospinning yielded a nanofibrous network with a very large surface area ratio (2140 m^2^ g^-1^) and high porosity allowing better nutrient/oxygen diffusion and enhancing osteoblast attachment and differentiation. Furthermore, this nanofiber exhibited good biocompatibility and mechanical properties, providing an optimal microenvironment for bone repair. Thus, Ni-MOF-based composites represent prospective advanced biomaterials for orthopedic applications 90.

Zhang *et al.* developed a Ni-MOF-based delivery system for treating postmenopausal osteoporosis. This system used Ni^2+^ to repair bone defects *in vitro*. Mechanistically, Ni^2+^ increased levels of VEGFA and key cell cycle proteins like Cyclin D1/D3, thereby supporting proliferation and neovascularization of HUVECs, providing nutrients, and attracting osteoprogenitors that could form new bone. The Ni-MOF system could also modulate aurora A kinase in macrophages to create an osteoimmune microenvironment important for repairing poor blood vessel growth in weak bones, offering a promising approach to repairing bone 91.

### 3.9 Tailoring Stem Cell Fate Regulation with Bimetallic MOF

**Figure 4:**
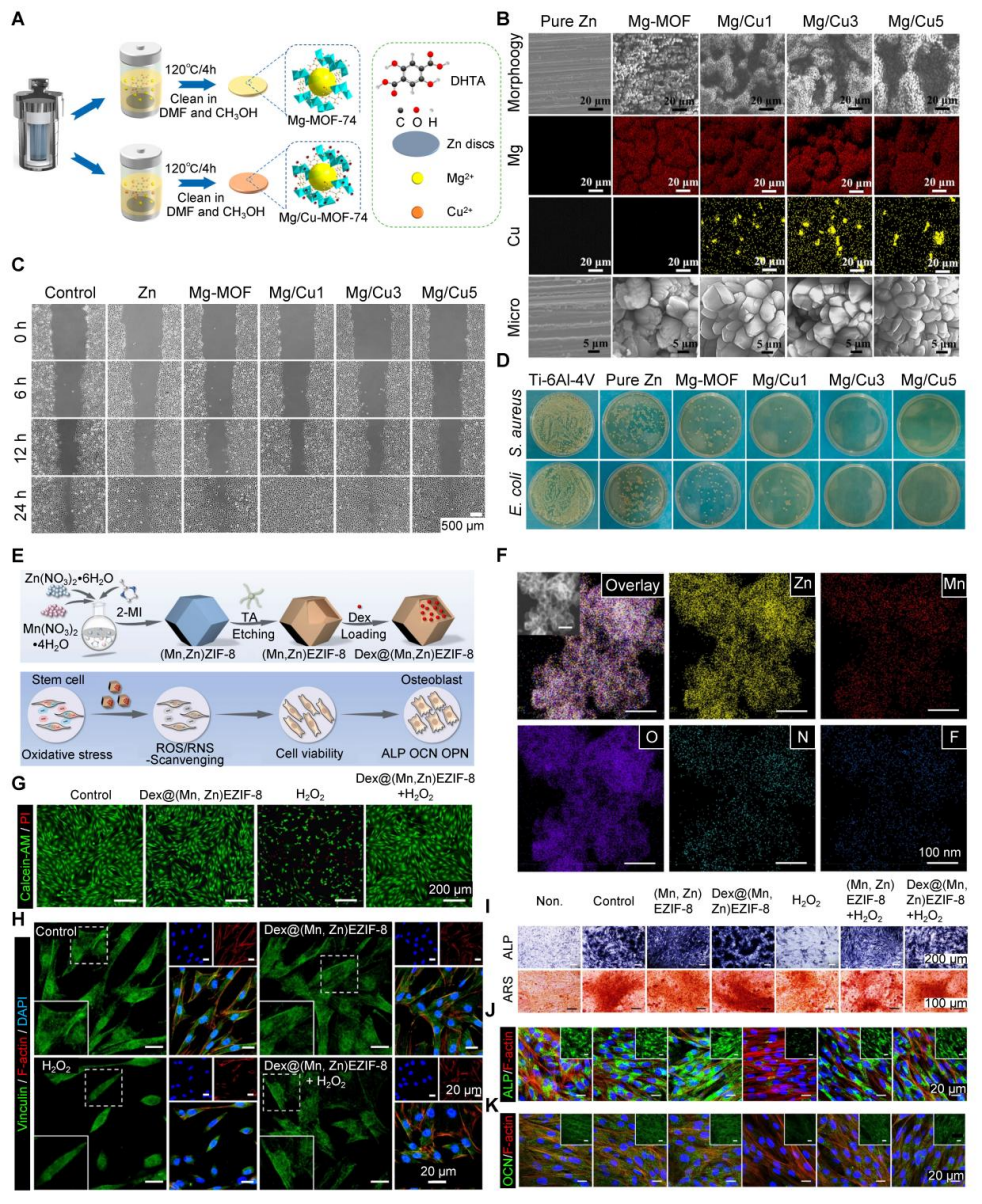
**Fabrication, characterization, and biofunctional assessment of MOF composites on Zn substrate. (A)** Schematic illustration showing the synthesis of Mg-MOF-74 and bimetallic Mg/Cu-MOF coatings made on a Zn substrate. **(B)** Surface characterization: representative SEM images and elemental mapping (Mg, Cu, Zn) of plain Zn, Mg-MOF, Mg/Cu1, Mg/Cu3, and Mg/Cu5 surfaces.** (C)** Migration assay *in vitro*: phase-contrast images showing the HUVECs migration after treatment with extracts from different alloy samples. **(D)** Antibacterial ability: CFU assay images showing antibacterial efficacy of the coatings against *S. aureus* and *E. coli*. **(E)** Diagram showing Dex@(Mn, Zn) EZIF-8 composite fabrication and the mechanism of protecting cells from ROS/RNS-induced damage. **(F)** Structural analysis: elemental mapping images of Dex@(Mn, Zn) EZIF-8 showing Mn, Zn, C, N, and O distribution. **(G)** Cell viability: live/dead staining (Calcein-AM/PI) on BMSCs under different treatments. **(H)** Osteogenic marker expression: immunofluorescence images of BMSCs stained for osteogenesis-related markers (*e.g.*, Runx2, OPN) after treatments. **(I)** Osteogenic differentiation quantification: ALP activity staining (day 7) and ARS mineralization staining (day 21) of BMSCs. **(J)** Dual immunofluorescence staining for ALP (green) and F-actin (red, phalloidin) in BMSCs. **(K)** Dual immunofluorescence staining for OCN (green) and F-actin (red) in BMSCs under different treatments. Adapted with permission from 92, copyright 2024 American Chemical Society; 93, copyright 2025 Royal Society of Chemistry.

Bimetallic MOFs offer multifunctional capabilities for bone regeneration by simultaneously mitigating oxidative stress and regulating stem cell metabolism. Chen *et al*. made a Mg/Cu bimetallic MOF coating on Zn-based membranes for bone repair. This bioactive membrane improved new bone formation, blood vessel growth, and antibacterial activity **(Figure 4A, B)**. By adjusting the Cu^2+^ doping level, the degradation behavior and ion release profiles of the membrane were precisely controlled. Upon degradation, Zn^2+^, Mg^2+^, and Cu^2+^ were released simultaneously, creating an alkaline microenvironment that facilitated calcium phosphate deposition. Consequently, the Mg/Cu-MOF coating could improve osteogenesis in BMSCs, vascularization of HUVECs **(Figure 4C)**, and bactericidal activity **(Figure 4D)** *in vitro* and *in vivo* 92.

Another studyused manganese (Mn) to fabricate Dex@(Mn,Zn)EZIF-8 **(Figure 4E, F)** to enhance catalase-like ROS scavenging, and applied tannic acid etching for introducing reactive nitrogen species (RNS) scavenging ability. This combined antioxidant effect protected MSC viability **(Figure 4G)** and adhesion **(Figure 4H)** under oxidative stress. The porous hollow structure enabled long-term release of dexamethasone (DEX), thereby promoting bone regeneration by significantly upregulating osteogenic protein levels **(Figure 4I-K)**. Therefore, bimetallic MOFs serve as a versatile nanoplatform, combining control of redox balance with enhanced osteogenesis for better bone repair 93.

Mn is important in ECM formation and holds great promise in bone defect repair 94. In a recent study, MnO_2_@UiO-66(Ce) was synthesized by adding manganese dioxide (MnO_2_) into the nanoscale mesoporous channels of a Ce-based UiO-66 MOF, creating an integrated SOD/CAT cascade catalytic system: the Ce-O nodes in UiO-66 acted like superoxide dismutase (SOD) breaking down superoxide anions, while the adjacent MnO_2_ functioned like catalase (CAT), converting H_2_O_2_ into water and O_2_. This MnO_2_@UiO-66(Ce) cascade system alleviated oxidative stress and rescued osteogenesis of PDLCs under inflammatory conditions. At the molecular level, it increased mitophagy through the SIRT1-FOXO3-BNIP3 signaling pathway, cleared damaged mitochondria, prevented mitochondrial ROS bursts, and restored cell homeostasis, thereby promoting bone repair 95.

In summary, MOFs promote bone healing as a useful reservoir of therapeutic ions. The long-lasting and localized release of bioactive metal ions (*e.g.*, Mg^2+^, Zn^2+^, Cu^2+^, Sr^2+^) initiates key cellular processes and signaling pathways that enhance osteogenesis, angiogenesis, and modulate the immune microenvironment. Furthermore, MOFs can be engineered as sophisticated nanozymes that scavenge ROS, thereby alleviating oxidative stress and breaking the inflammatory cycle that impedes healing. By combining osteoinductive, angiogenic, immunomodulatory, and antioxidant properties in a single platform, MOF-based biomaterials create a great microenvironment that accelerates bone defect repair.

## 4. MOFs as Advanced Delivery Carriers

MOFs, with their large surface area and tunable porosity, represent powerful platforms for drug delivery. The drugs can be loaded through methods like adsorption, encapsulation, and covalent or non-covalent functionalization. This feature is particularly advantageous for stabilizing shorthalf-life drugs and enabling localized, long-lasting release, thereby boosting treatment efficacy while minimizing systemic toxicity. Furthermore, the structural and chemical flexibility of MOFs allows for the design of stimuli-responsive and targeted delivery systems capable of detecting and treating pathological tissues with high precision 96. For bone healing, scaffolds with drugs or signaling molecules from MOFs have the potential to enhance osteoblast proliferation and differentiation, significantly improving the therapeutic ability of MOFs as advanced delivery carriers 97.

### 4.1. Proteins and Small Molecules: Stabilization and Sustained Release of Osteoinductive Factors

A main use of MOFs is to deliver osteogenic proteins to support bone growth. These proteins are important for bone repair but have issues such as short half-life, instability, and cause ectopic ossification when given systemically. MOFs solve these problems by creating a safe microenvironment that prevents rapid degradation and enables controlled, local release.

Toprak *et al.* incorporated BMP-6-loaded ZIF-8 nanoparticles into an electrospun polycaprolactone (PCL) membrane. This PCL/BMP-6@ZIF-8 composite achieved ~98% loading efficiency and could slowly release BMP-6 for more than 30 days. In a calvarial defect model, this composite increased bone volume by about 17%, which was 7% higher than the control PCL membrane. These results showed that MOF carrier systems help stabilizeand deliver delicate biological materials to enhance bone healing 98.

MOFs are also useful for targeted delivery of certain small-molecule drugs like DEX, simvastatin, and antibiotics. These drugs help treat conditions such as osteoporosis, impaired bone healing, and infected bone defects. Normally, these drugs suffer from poor solubility, short half-life, and dose-limiting systemic side effects when given conventionally. MOFs help solve these issues because of their high drug-loading capacity and tunable degradation kinetics that allow controlled release as needed 99.

Liang *et al.* constructed a bioinspired system by using stem cell membranes (SCM) to encapsulate DEX-loaded ZIF-8 nanoparticles 100. The SCM coating helped reduce immune reactions and allowed better targeting to BMSCs. This DEX@ZIF-8-SCM composite allowed high DEX loading and controlled intracellular release. It significantly improved bone formation by activating the PI3K-Akt signaling pathway and upregulating genethe expression of genes such as *Osterix* and *Smad4*. In another study, Shen *et al.* developed a multifunctional coating for Ti implants by combining Zn-based MOFs with raloxifene (Ral), a selective estrogen receptor modulator used for osteoporosis 101. The MOF coating slowly released Ral and Zn^2+^, synergistically helping to treat osteoporosis. This approach showed the potential of MOFs to deliver bioactive proteins and small molecules beyond conventional drugs.

### 4.2. Integration of MOFs with Extracellular Vesicles

Despite their many advantages, MOFs also face translational challenges such as limited targeting capability and a foreign body response. However, a combination of MOFs and extracellular vesicles (EVs) could be a good solution. EVs have the merits of innate immune evasion and homologous targeting, while MOFs offer stability and controlled release kinetics. This combined system also allows the simultaneous loading of multiple therapeutic agents, making it useful for complex tissue repair. This powerful combination shows great promise for solving complex challenges in regenerative medicine 102.

**Figure 5:**
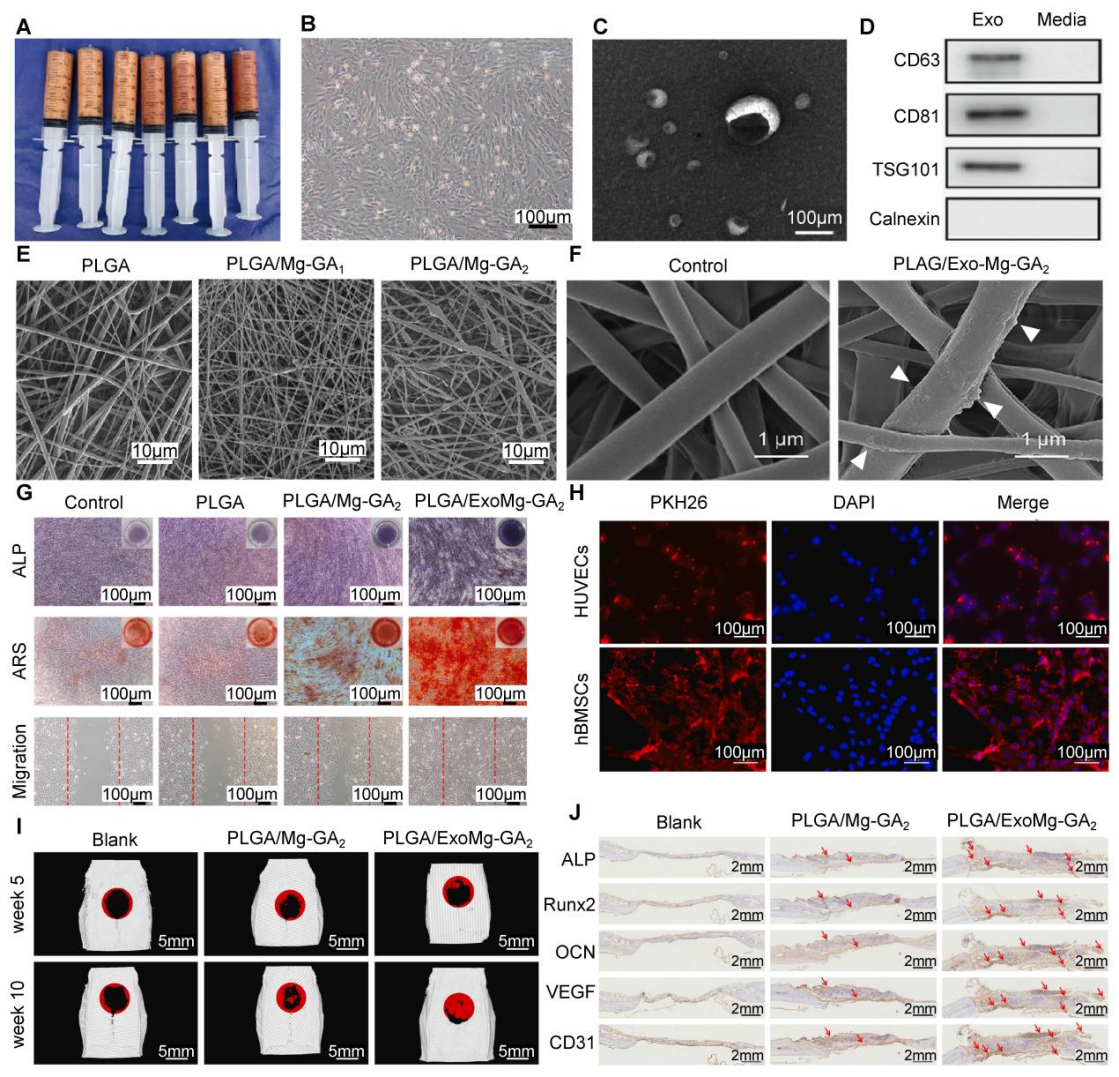
** Use of hADSC exosomes for bone regeneration. (A)** Images of human adipose tissue used for stem cell isolation. **(B)** Cell morphology of hADSCs. **(C)** Representative SEM images of exosomes isolated from hADSC conditioned medium. **(D)** Western blot analysis confirming the presence of exosomal-positive markers (CD63, CD81, TSG101) and the absence of the negative marker (Calnexin). **(E)** Surface morphology: representative SEM images showing the surface topography of each fabricated sample group. **(F)** Exosome coating: SEM images showing the surface of pure PLAG/Mg-GA_2_ with and without exosomes. **(G)** *In vitro* functional tests: ALP staining, ARS staining, and migration assay. **(H)** Cellular uptake tracking: immunofluorescence images of HUVECs and hBMSCs stained with PKH26-labeled exosomes (red) to evaluate internalization. **(I)** *In vivo* evaluation: micro-CT reconstructions of critical-sized bone defects, treated with different samples. **(J)** Histological staining for key osteogenic markers (ALP, Runx2, OCN) and angiogenic markers (VEGF, CD31). Adapted with permission from 33, copyright 2022 KeAi.

Native EVs, such as those derived from human adipose-derived stem cells (hADSCs), have a short half-life and are prone to rapid degradation 103. To overcome this, Kang *et al.* collected hADSC-derived EVs **(Figure 5A-D)**, and incorporated them into poly(lactic acid-co-glycolic acid) (PLGA) with Mg-GA MOF **(Figure 5E, F)**. This mix made a stable structure with slow-release features, along with the innate targeting and immune-evasive properties of EVs. ALP and ARS staining, and migration assay showed that the scaffold greatly promoted osteogenesis of hBMSCs **(Figure 5G)** and enhanced angiogenesis of HUVECs** (Figure 5H)**. *In vivo,* it accelerated bone remodeling and improved osseointegration **(Figure 5I, J)**, showing strong promise for medical use 33.

### 4.3. Nucleic Acids: Developing Gene-Activated Matrices for Targeted Pathway Regulation

Gene-activated matrices are being advanced for the repair of bone defects 104. These materials release nucleic acids (*e.g.*, plasmid DNA, siRNA, and miRNA) in a controlled way. This helps to manage bone formation by upregulating Runx2 expression or blocking negative regulators like miR-138. This approach can direct cell differentiation and bone regeneration. But using gene therapy in patients poses crucial challenges, including instability, limited cellular uptake, and endosomal degradation of nucleic acids.

MOFs offer a promising non-viral delivery platform due to their high loading capacity and superior protection against enzymatic degradation. Feng *et al.* showed the feasibility of this approach by co-loading miR-21 (pro-angiogenic) and miR-5106 (pro-osteogenic) into ZIF-8 nanoparticles using a simple one-step method. These were easily taken up by cells, solving problems that standard delivery systems have. RNA sequencing of HUVECs treated with miR-21@ZIF-8 showed activation of MAPK/HIF-1 signaling pathways, both critical for angiogenesis, highlighting the positive effects of MOF-based co-delivery systems for tissue repair 105.

### 4.4. Stimuli-Responsive Release Systems

Stimuli-responsive MOF-based release systems are advancing significantly toward precision medicine in bone defect repair. These systems can be engineered to deliver and release therapeutic factors in response to specific internal signals (*e.g.*, pH) or external triggers (*e.g.*, light, ultrasound), enabling spatiotemporal control over the physiochemical properties of MOF, modulating the therapeutic effects, and eliminating the side effects.

**4.4.1 pH-Responsive Systems:** Acidic microenvironments characteristic of bone resorption sites, bacterial infections, or tumor tissues allow targeted drug release from pH-sensitive MOFs such as ZIF-8. An innovative acid-responsive ZIF-8 system (ZNC) that included sodium bicarbonate and RANKL-CRISPR/Cas9 plasmids effectively neutralized the acidic microenvironment, improved transfection efficiency, inhibited osteoclast formation, and promoted osteogenic differentiation and mineralization in ovariectomized mouse models 106. Similarly, Shen *et al.* constructed a bone-targeted nanocarrier (CZ@HA/ALN) functionalized with hyaluronic acid and alendronate, achieving a 3.3-fold higher curcumin release at pH 5.0 than at pH 7.4, thereby improving antitumor efficacy in tibial metastasis 107.

**Figure 6:**
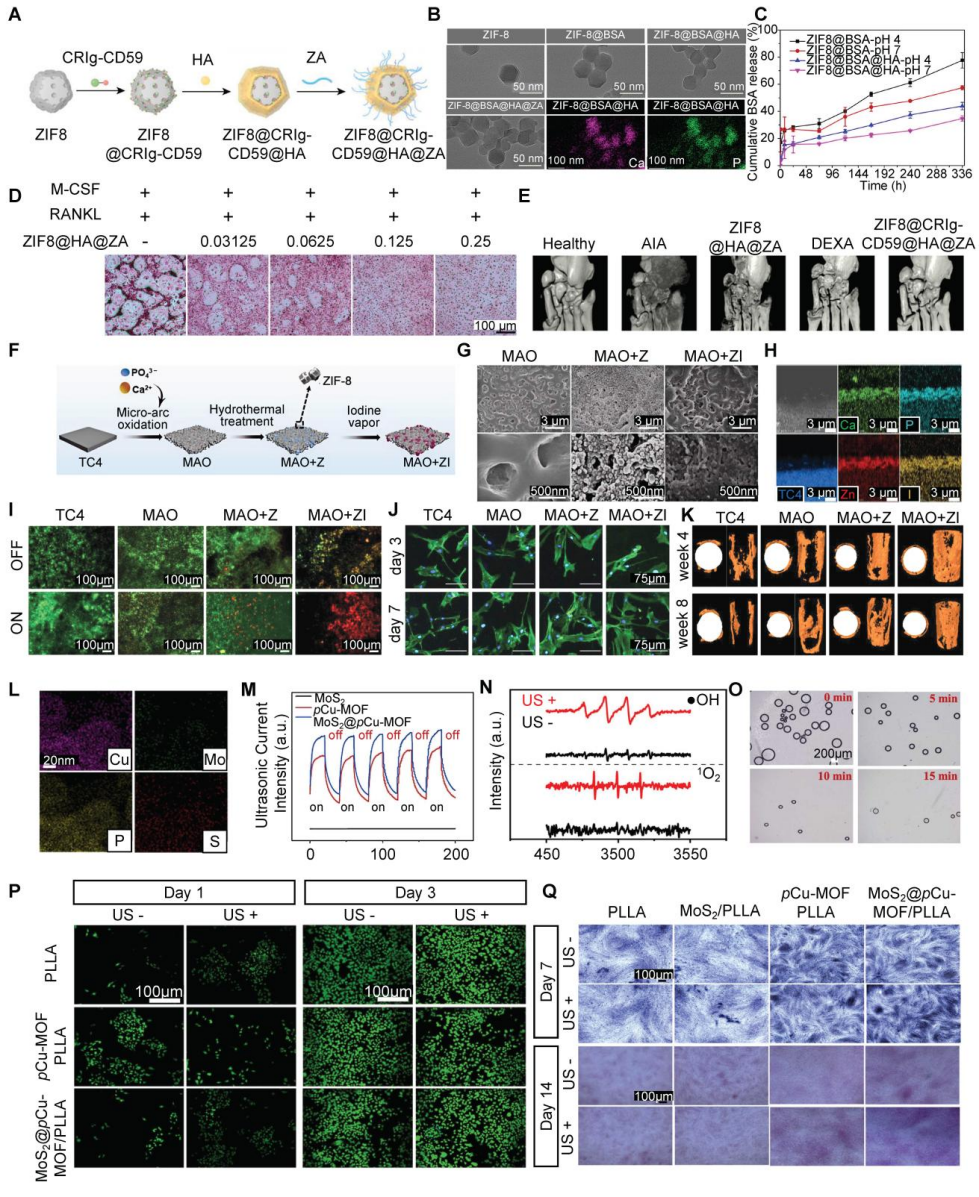
** Multifunctional nanocomposite and coating systems for therapeutic delivery and bone regeneration. (A)** Fabrication schematic of ZIF8@CRIg-CD59@HA@ZA. **(B)** TEM images and Ca and P elemental mapping of nanoparticles. **(C)** pH-dependent BSA release profiles from ZIF8@BSA and ZIF8@BSA@HA NPs. **(D)** Trap staining showing ZIF8@HA@ZA inhibits osteoclastogenesis. **(E)** Micro-CT images of ankle joints in an arthritis model. **(F)** Fabrication schematic of the iodine-loaded MAO+ZI coating. **(G)** SEM images of MAO, MAO+Z, and MAO+ZI coatings. **(H)** Elemental mapping of MAO+ZI. **(I)** Live/dead staining of *S. aureus* and *E. coli* biofilms on samples.** (J)** Cytoskeleton (F-actin) and nuclei (DAPI) of BMSCs on coatings. **(K)** New bone formation around implants *in vivo*.** (L)** Elemental mapping of MoS_2_@pCu-MOF. **(M)** Ultrasonic current of MoS_2_, pCu-MOF, and MoS_2_@pCu-MOF. **(N)** EPR spectra confirming US-activated ROS generation by MoS_2_@pCu-MOF/PLLA. **(O)** Ultrasound-triggered O_2_ bubble generation from MoS_2_@pCu-MOF. **(P)** Live/dead staining of BMSCs on days 1 and 3. **(Q)** ALP (day 7) and ARS (day 14) staining of BMSCs. Adapted with permission from 108, copyright 2023 American Chemical Society; 113, copyright 2021 Wiley; 115, copyright 2025 Elsevier.

Tao *et al.* fabricated a pH-responsive ZIF-8-based nanoplatform encapsulating CRIg-CD59 and surface-mineralized with zoledronic acid (ZA) for rheumatoid arthritis therapy **(Figure 6A, B)**. In the acidic microenvironment of inflamed joints, ZIF-8 dissociated, releasing CRIg-CD59 **(Figure 6C)** to inhibit complement activation, alleviating inflammation and protecting tissue. Simultaneously, the released ZA inhibited osteoclast-mediated bone resorption **(Figure 6D)**, collectively restoring the synovial macrophage niche and promoting joint repair **(Figure 6E)** 108.

**4.4.2 Microenvironment-Responsive Systems:** A Ce/Sr-based bifunctional MOF exemplifies an advanced microenvironment-responsive design that actively modulates the bone microenvironment to promote regeneration. This MOF releases Ce and Sr ions in response to the acidic and oxidative conditions typical of osteoporotic bone. Its intrinsic SOD and CAT-like catalytic activities decrease mitochondrial ROS, restore mitochondrial function, enhance mitophagy, and rebalance mitochondrial dynamics by suppressing fission while promoting fusion. Sr ions further support osteogenic differentiation, while bisphosphonate ligands inhibit osteoclast activity. This synergistic, microenvironment-responsive mechanism coordinates response reprogramming of senescent MSCs, reactivates osteoblastogenesis, and facilitates robust osseointegration, providing a targeted therapeutic strategy for bone defect repair 109.

Yang *et al.* fabricated a thermo-sensitive injectable hydrogel (SFD/CS/ZIF-8@QCT) incorporating quercetin-modified ZIF-8 nanoparticles which exhibit excellent pH sensitivity, enabling intelligent and sustained release of zinc ions and quercetin specifically within the acidic periodontitis niche. The system addresses multiple therapeutic challenges: it provides antibacterial activity, rapid hemostasis, macrophage reprogramming from M1 to M2, and improved osteogenic/angiogenic differentiation ability of PDLSCs. Transcriptomic analysis verified its regenerative effects, which are regulated by activation of the PI3K-Akt pathway, restoring cellular metabolism, reducing oxidative stress, and inhibiting excessive autophagy. This multi-functional, pH-triggered hydrogel exemplifies an advanced biomaterial strategy for comprehensive periodontal tissue engineering 110.

**4.4.3 Light-Responsive Systems:** Near-infrared (NIR)-responsive MOF composites enable externally controlled photothermal and photodynamic therapy. Yang *et al.* constructed a ZIF-8/graphene oxide (GO) composite that generated localized hyperthermia under NIR irradiation, disrupting bacterial biofilms while releasing antibacterial Zn^2+^ 111. Liu *et al.* designed a ZIF-8-PDA-HA nanosystem for osteoarthritis treatment, which released diclofenac sodium under NIR irradiation, improved joint lubrication, and upregulated chondrogenic markers (Col2α and Acan)^112^.

Teng *et al.* fabricated a light-responsive bactericidal composite with ZIF-8 immobilized with iodine (MAO+ZI) on titanium implants **(Figure 6F-H)**. The composite coating exhibited excellent NIR-triggered properties, enabling controlled “burst” release of iodine upon 808 nm laser irradiation. This on-demand release is attributed to differential light absorption between ZIF-8 and the substrate, causing localized thermal expansion and structural dissociation. Simultaneously, NIR irradiation activated ZIF-8 to generate singlet oxygen (^1^O_2_), synergizing with the released iodine to produce substantial intracellular ROS, effectively disrupting bacterial membranes and eradicating biofilms **(Figure 6I)** without compromising biocompatibility **(Figure 6J)**. Additionally, the system improved osteogenic differentiation and osseointegration. This dual-functional, light-triggered strategy offers a promising approach to combat implant-associated infections while promoting bone repair **(Figure 5K)** 113.

**4.4.4 Ultrasound-Responsive Systems:** Ultrasound (US)-responsive MOFs enable deep tissue sonodynamic therapy (SDT). Yu *et al*. designed a porphyrinic MOF coated with red blood cell membranes, achieving a 99.9% antibacterial activity against methicillin-resistant *S. aureus* (MRSA) upon US exposure, providing a promising treatment for osteomyelitis 114.

Pan *et al.* fabricated a US-responsive MoS_2_@pCu-MOF heterojunction scaffold designed for synergistic antibacterial activity and bone regeneration **(Figure 6L)**. Under US activation, the type-II heterojunction between MoS_2_ and phosphate-based Cu-MOF facilitated electron-hole separation, thereby significantly enhancing ROS generation, including •OH and ^1^O_2_, independent of the H_2_O_2_ level **(Figure 6M-O)**. This sonodynamic effect increased bacterial membrane permeability, enabling deep penetration and effective pathogen eradication*.* Moreover, the US-induced microcurrent, in synergy with PO_4_^3-^ release from pCu-MOF, promoted osteogenesis by increasing the levels of Runx2, BMP2, and Wnt10b, enhancing bone formation by up to 36% in late-stage osteogenesis. This work highlights the dual-mode therapeutic potential of US-activated MOF scaffolds in treating infected bone defects through combined antibacterial and osteoinductive actions **(Figure 6P, Q)** 115.

With the rapid advancement of biomedical engineering, MOFs have become a versatile platform for controlled delivery of diverse bioactive agents in bone tissue engineering. Their high surface area and tunable porosity enable efficient loading and protection of therapeutic agents, including osteogenic proteins, small molecules, and nucleic acids. Stimuli-responsive designs allow precise, on-demand release in pathological microenvironments or in response to external triggers. This spatiotemporal control ensures high local biocompatibility while minimizing systemic side effects. By integrating controlled release with inherent osteoinductive, angiogenic, immunomodulatory, and antibacterial properties, MOF-based systems orchestrate multiple regenerative processes. These intelligent, multifunctional delivery platforms hold significant promise for solving current challenges in bone defect repair.

## 5. MOF-Integrated Composite Scaffolds for Synergistic Bone Repair

The integration of MOFs into composite scaffolds has made significant progress in bone regeneration 116. These nanocomposites with MOFs are classified into several groups: bio-MOFs (designed for optimal interaction with biological systems), metal MOFs (incorporating metallic elements to improve mechanical and biological performance), non-metal MOFs (utilizing non-metallic components to modify material properties), and semiconductor MOFs (used in photothermal therapy and photocatalytic applications). Each group provides different benefits for bone defect repair, promoting the development of optimized biomaterials 117.

### 5.1. MOF-Polymer Hybrid Systems

Although polymers are known for their biocompatibility, their use in bone regeneration has been limited by poor osteogenic efficiency, weak mechanical stiffness, and stability. Incorporating MOFs into polymer matrices can be a promising strategy, which combines the complementary strengths of both materials 118. This synergistic approach addresses the main problems of conventional hydrogels while improving MOF processability and stability. Mixed materials perform better by continuously releasing ions, being structurally stable, and exhibiting better biological activity 119.

**Figure 7:**
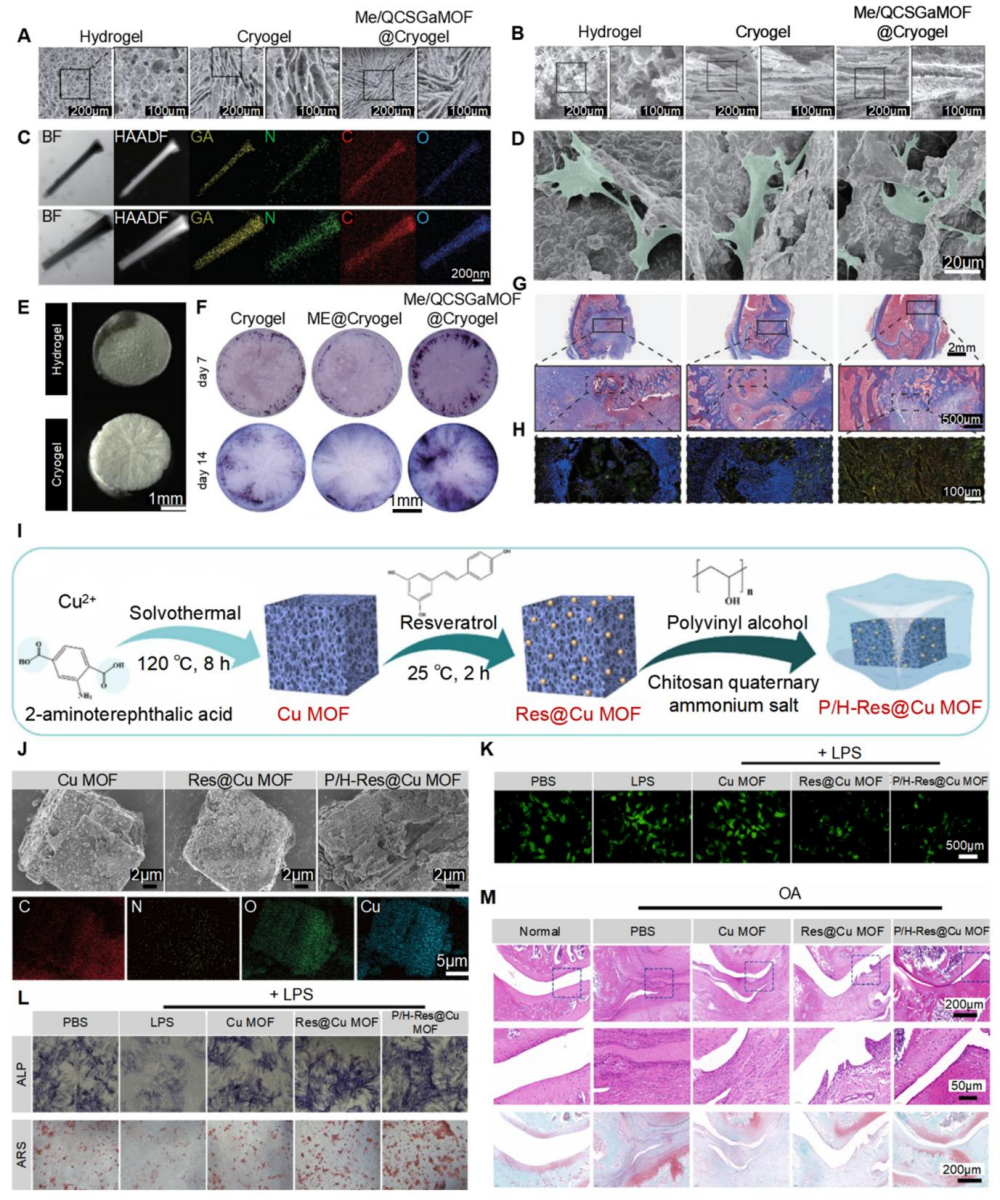
** Advanced cryogel and MOF scaffolds for bone repair. (A)** Surface SEM images showing porosity of hydrogel, cryogel, and Me/QCSGaMOF@Cryogel. **(B)** Cross-sectional SEM images showing the macroporous structure inside scaffolds. **(C)** Elemental mapping of Ga distribution in GaMOF and QCSGaMOF. **(D)** TEM images showing cell adhesion and spreading morphology on the scaffold. **(E)** Macroscopic images of each scaffold. **(F)** ALP staining of early osteogenesis. **(G)** Masson's trichrome staining of infected femoral defects. **(H)** Immunofluorescence staining for OCN (yellow) and LEPR (green) in defect sites. **(I)** Fabrication process of polydopamine/hyaluronic acid-resveratrol@Cu MOF composite. **(J)** SEM images with elemental mapping (Cu, C, O, N) of P/H-Res@Cu MOF composite surfaces. **(K)** Fluorescence staining of intracellular ROS using DCFH-DA probe. **(L)** ALP activity (day 7) and ARS staining (day 21). **(M)** Histological evaluation using H&E staining and SO/FG staining of bone/cartilage matrix *in vivo*. Adapted with permission from 120, copyright 2023 Wiley; 123, copyright 2021 Elsevier.

Liu *et al.* developed a radially oriented cryogel using directional freeze-casting **(Figure 7A & B)**. They added methicillin- and quaternized chitosan-modified gallium MOFs (Me/QCSGaMOF) **(Figure 7C)** in the cryogel, which has an oriented structure that guides the ingrowth of bone cells **(Figure 7D & E)** and increases osteogenesis by BMSCs **(Figure 7F)**. This process involves the activation of the Wnt/β-catenin signaling pathway. *In vivo* results confirmed it could clear infections and guide new bone formation at the infected site **(Figure 7G & H)**, demonstrating its dual function in clearing infections and repairing bone 120.

Another study developed an injectable hydrogel composed of catechol-chitosan (CA-CS) modified with ZIF-8 and found that a 1.2 mg dose of CA-CS/ZIF-8 hydrogel significantly enhanced bone formation in a rat skull defect model. This treatment reached a bone volume to total volume (BV/TV) ratio of 22.95% ± 2.39%, which is a 1.5-fold increase over the pure CA-CS hydrogel and a 2.7-fold increase compared to the control group 121.

In another study focusing on mechanical enhancement, Qiao *et al.* made a simvastatin-loaded ZIF-8 (SIM@ZIF-8) composite, which they dispersed within a mixed matrix of poly(ethylene glycol) diacrylate (PEGDA) and sodium alginate (SA). This nSZPS hydrogel could steadily release simvastatin for 21 days (cumulative releases of 68.5% at pH 7.4 and 80.3% at pH 5.5). *In vitro* and *in vivo* evaluations showed improved bone regeneration *in vivo*, with a remarkable BV/TV ratio of 52.6% compared to 15.3% in controls 122.

Huang *et al.* designed a bionic PVA/HACC-coated Cu-based MOF hydrogel (P/H-Res@Cu MOF) to treat osteoarthritis (OA) **(Figure 7I)**. This hydrogel helped with lubrication, reduced frictional damage to cartilage **(Figure 7J)**, and released resveratrol and Cu^2+^ in the acidic OA microenvironment. In addition, these components could form metal-polyphenol chelates, enhancing antioxidant activity **(Figure 7K)**, promoting macrophage M2 polarization, and increasing osteogenesis **(Figure 7L)**. These biological functions together repaired the damaged bone and cartilage for OA **(Figure 7M)** 123. Similarly, Moris *et al.* used the freeze-drying method to incorporate Zr-based MOF-801 into a gelatin matrix. This composite not only promoted apatite formationunder simulated body fluid conditions, but also showed good biocompatibility. Its sustained release of Zr ions and fumarate further enhanced mineralization in MG-63 cells, thus exhibiting great potential for bone tissue engineering 124.

Polymers such as polycaprolactone (PCL), polyvinyl alcohol (PVA), and PLGA are widely investigated bioactive electrospun fibers, but their use for bone repair is often limited. Xue *et al.* modified PCL/collagen (PCL/Col) fibers with ZIF-8 using a post-electrospinning hydrothermal technology. This PCL/Col/ZIF-8 composite with a controlled and sustained Zn^2+^ release ability significantly enhanced osteogenesis and angiogenesis compared to PCL/Col controls 125. In a similar study, Ramezani *et al.* created electrospun polyacrylonitrile (PAN) fibers incorporating different concentrations of Fe(III)-MOF. The data showed that PAN loaded with 5 - 10% Fe-MOF had improved biocompatibility and great potential for promoting tissue regeneration *in vivo* 126.

New manufacturing technologies, such as 3D printing, enable the fabrication of biomaterials with precisely controlled structures. Using extrusion-based 3D printing, a composite structure was fabricated by incorporating ZIF-8 into a mixed matrix of dicalcium phosphate dihydrate (DCPD) and PCL. This structure showed strong mechanical strength and an interconnected porous architecture that continuously released Ca^2+^ and Zn^2+^, promoting BMSC proliferation and new bone formation 127.

Xia *et al.* created fiber structures with ZIF-8-based carbon nanoparticles (C-ZnO). These special nanostructures combined the topographical cues of carbon nanomaterials with the biological benefits of Zn^2+^, providing many binding sites for cell membrane receptors, while simultaneously inhibiting bacterial growth. These modified 3D printed structures improved cell spreading and increased expression of osteogenic markers (ALP, IBSP, and vinculin), boosting bone repair through combined physical and chemical cues 128.

In summary, MOF-polymer composites offer a versatile, multifunctional platform for bone regeneration, successfully overcoming the limitations of individual components. The diverse fabrication strategies, hydrogels, electrospun fibers, and 3D printed constructs consistently demonstrate enhanced mechanical properties, sustained therapeutic release, improved osteogenic activity, and potent antibacterial effects across multiple systems. These advanced composites also orchestrate bone regeneration through controlled ion release and topographic cues, positioning them as next-generation solutions for challenging bone defects. Future efforts will aim to optimize release kinetics, improve integration with host tissues, and develop smart, physiologically responsive systems.

### 5.2. MOF-Bioceramic and Implant Composites

Integrating MOFs with bioceramics is a smart way to engineer scaffolds that combine ceramic stiffness with MOFs' bioactive features. Regular bioceramics, including hydroxyapatite (HA) and β-tricalcium phosphate (β-TCP), are often used to repair bone defects because of their good osteoconductivity and compositional similarity to natural bone. However, they lack controlled-release capability to actively modulate biological response. But MOF incorporation can offer sustained drug delivery, therapeutic ion release, and stimuli-responsive behavior, while preserving the favorable mechanical properties of the ceramics. This combination creates a new class of intelligent bone repair materials that provide both structural support and achieve specific clinical aims in a spatially and/or temporally controlled manner.

Building on this foundation, Sarkar *et al.* made a 3D cellulose-HA nanocomposite incorporating DEX-loaded MOF (HA/DMOF). This composite had 60-80 nm DMOF nanoparticles and showed stiffness similar to cancellous bone. An important advance was the extended-release formulation of DEX over 4 weeks, which was longer than DMOF alone. The HA/DMOF scaffold showed good biocompatibility with pre-osteoblasts and increased alkaline phosphatase activity and mineralization, demonstrating its potential as a useful solution for orthopedic applications 129.

**Figure 8:**
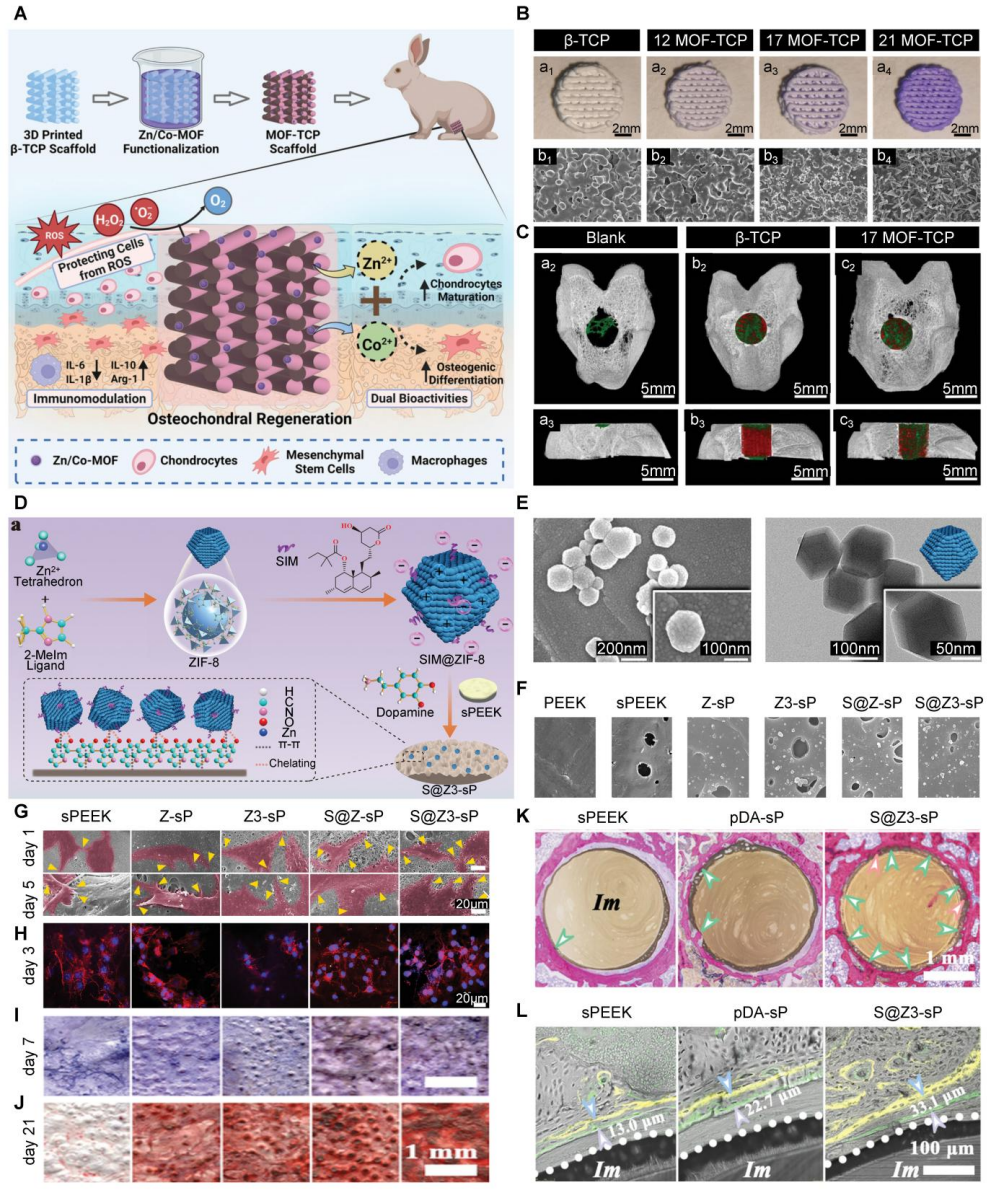
** 3D Printed and surface-modified MOF composites for OC and bone regeneration. (A)** Diagram of 3D printed MOF-functionalized tricalcium phosphate (MOF-TCP) scaffold. **(B)** Morphological and SEM images of scaffolds.** (C)** 3D reconstruction images of the defective bone. **(D)** Diagram of sulfonated ZIF-8 modified PEEK composite (S@Z3-sP). **(E)** SEM and TEM images characterizing the morphology and crystallinity of synthesized ZIF-8 nanoparticles.** (F)** SEM images of changes in PEEK implant topography. **(G)** SEM images of osteoblasts cultured on sample surfaces, with cellular pseudopodia indicated by yellow arrows. **(H)** Fluorescence images of cytoskeleton (F-actin) and nuclei (DAPI) showing cell adhesion and spreading. **(I)** ALP staining (day 7). **(J)** ARS staining (day 21). **(K)** H&E-stained histological sections of bone-implant interface; newly formed bone contacting implant surface (green arrows) and bone ingrowth into the implant pores (pink arrows). **(L)** Dual fluorescence labeling (calcein/alizarin red) of newly regenerated bone around implants; inter-label distance indicates mineralization rate. Adapted with permission from 130, copyright 2023 Wiley; 133, copyright 2021 Wiley.

Other bioceramics apart from HA have also been successfully modified with MOFs to enhance their therapeutic potential **(Figure 8A)**. Shu *et al.* created a 3D-printed β-TCP scaffold modified with a bimetallic Zn/Co-MOF for treating OC defects **(Figure 8B)**. This composite provided structural support while showing anti-inflammatory and ROS-scavenging capabilities. It was effective in addressing the complex conditions in OA and promoting subchondral bone repair **(Figure 8C)** 130. MOF-bioceramic composites were also applied to the surface of orthopedic implants to improve biointegration. Li *et al.* fabricated a ZIF-8-modified alkali and heat-treated Ti (ZIF-8@AHT), which enhanced osteogenic capacity by promoting osteogenic gene expression, ECM formation, and mineralization. The porous ZIF-8 structure allowed drug loading, while the sustained release of Zn^2+^ further conferred angiogenic, antibacterial, and hemostatic properties. At the molecular level, ZIF-8 promoted osteogenesis by facilitating cellular uptake and therefore activating the MAPK signaling pathway in BMSCs 131.

PEEK is often used in bone tissue engineering because of its outstanding mechanical properties, chemical stability, and good biocompatibility. Of note, PEEK's elastic modulus is close to that of bone, making it suitable for load-bearing implants 132. But, PEEK is inherently bio-inert, thus requiring surface modifications to enhance its osteointegration. Deng *et al.* made a heterostructured coating of simvastatin-loaded ZIF-8 **(Figure 8D, E)** and polydopamine on the surface of porous PEEK **(Figure 8F)**. This modified PEEK showed strong osteointegration *in vitro* through Zn^2+^ release, drug delivery, topological cues **(Figure 8G, H)**, and osteogenic potential **(Figure 8I, J).** When exposed to NIR, the coating can release heat, ^1^O_2_, and Zn^2+^, enabling effective photothermal/photodynamic antibacterial activity. Both *in vitro* and *in vivo* results verified its photo-switchable disinfection and superior osseointegration **(Figure 8K, L)**, highlighting its great promise for treating infected bone defects 133. Xiao *et al.* made a novel Zn/Mg-MOF74 coating on PEEK implants. They first applied polydopamine and then used the hydrothermal method to create a uniform MOF coating (PEEK-74), which they loaded with DEX to form PEEK-DEX. The modified PEEK showed enhanced antibacterial activity due to the synergistic effect of both ion and drug delivery. More importantly, *in vivo* evaluation showed that PEEK-DEX significantly promoted bone healing, demonstrating the potential of MOF-based coatings to transform bio-inert polymers into bioactive implants that are capable of supporting osseointegration 134.

In summary, MOF-bioceramic composites combined the structural stability and osteoconductivity of bioceramics and the bioactivity of MOFs. These hybrid composites solve key problems of conventional bone grafts by enabling controlled release of therapeutic agents, enhancing bioactivity, and improving integration with host tissue. Future research should aim to fine-tune release kinetics, improve mechanical properties, and develop smart systems that respond to physiological signals, ultimately paving the way for a new generation of smart bone-repair biomaterials.

### 5.3. Synergistic Interactions with Other Emerging Materials

The versatility of MOFs can be further enhanced by integrating them with advanced biomaterials, creating synergistic systems that address multiple challenges in bone defect repair. These hybrid systems not only promote MOF functionality but also introduce new capabilities, such as improved biocompatibility, targeted delivery, and enhanced bioactivity.

Nanozymes, which simulate the catalytic activity of natural enzymes, can be integrated with MOFs to create multifunctional platforms. For instance, Ce-based UiO-66 exhibits SOD-like activity, which can be combined with CAT-mimicking nanozymes, such as MnO_2_, to form cascade catalytic systems. MnO_2_@UiO-66(Ce) efficiently scavenges mitochondrial ROS, activates mitophagy via SIRT1-FOXO3-BNIP3 signaling, and restores cellular homeostasis and osteogenesis in PDLCs. These integrated nanozyme-MOF systems provided immediate ROS clearance and long-term mitochondrial regulation, making them highly promising for treating inflammatory bone defects, where oxidative stress and mitochondrial dysfunction are key pathogenic factors 95.

Feng *et al.* developed Cu-MOFs with dual SOD- and CAT-like activities, incorporated into a pH-responsive oxidized dextran and dopamine-gelatin hydrogel. This system effectively eliminated bacteria, modulated the immune microenvironment, promoted angiogenesis, and supported cell viability and osteogenesis 135.

MOFs with Cell membrane-coated MOFs create biomimetic nanoplatforms with prolonged circulation, immune evasion, and precise targeting capabilities. Jiang *et al.* encapsulated a miRNA-loaded ZIF-8 MOF core with a genetically engineered stem cell membrane overexpressing the CXCR4 receptor (CM-miR-21-m@MOF). This design endowed the nanoparticles with bone-targeting and ischemia-guiding capabilities by exploiting the natural CXCR4-SDF1 chemotactic axis 136. The biomimetic coating enabled active homing of nanoparticles to the ischemic femoral head *in vivo*, dramatically improving miRNA delivery for osteonecrosis therapy.

Similarly, Peng *et al.* designed a hollow ZIF-8 MOF loaded with polyphyllin II and cloaked with MSC membranes, forming the PZ@M-T platform. The MSCm coating provided prolonged *in vivo* retention and targeted delivery 137. In another study, Feng *et al.* developed Cu-MOF-based nanozymes with dual SOD- and CAT-mimicking activities for efficient ROS scavenging. Incorporated into a pH-responsive hydrogel that consisted of oxidized dextran and dopamine-functionalized gelatin. This system released biomimetic nanozymes into the acidic microenvironment of inflammatory bone defects^138^.

These synergistic systems collectively eliminate ROS and bacteria, modulate immunity, sustain stem cell viability, and promote osteogenesis and angiogenesis, creating a highly supportive microenvironment for bone repair.

### 5.4. Mechanical Properties and Load-Bearing Potential

Although MOF-polymer or MOF-ceramic composites offer strong bioactivity and controlled release, a critical clinical question remains: can these materials meet the mechanical requirements of load-bearing bone? Natural cortical bone exhibits remarkable mechanical stiffness, with a compressive strength of 130-180 MPa and an elastic modulus of 10-30 GPa 139. However, most MOFs are not mechanically strong, and are usually used as functional additives instead of structural components 140.

Mechanical improvements in MOF composites have to depend on the base material. For instance,

a) Incorporation of SIM@ZIF-8 into a PEGDA/SA hydrogel increased its compressive strength to approximately 1 MPa, representing a 1.6-fold increase over the ZIF-8-free controls 122. This improvement is mainly useful in very low-load environments or for soft-tissue encapsulation.

b) Zr-MOF-801 in gelatin scaffold can reach15 MPa approaching close to the strength of trabecular bone, but still not enough for heavy-weight-bearing^128^.

c) Choi *et al.* made a Ca/Mg MOF-loaded GelMA hydrogel for calvarial bone defect repair, which is a non-load-bearing application. Their study showed that the MOF-composite hydrogel can preserve structural integrity and viscoelastic properties well; these features are especially well-suited for repairing craniofacial or maxillofacial bone, where biological activity and controlled ion release are more critical than load-bearing capacity 141.

These studies highlight a fundamental issue: many MOF-composite strategies achieve only slight improvements from a very soft material, which is still not suitable for fixing bones that need to support weight. Another major limitation is that there are very few studies examining how their strength changes over time during degradation. For example, the slow release and degradation may cause holes to form inside the polymer matrix, making it fragile and posing risks for medical use. Therefore, a major design change is needed to enable MOF composites to meet the mechanical demands of load-bearing bone repair. Future strategies should focus on structural hybridization and interface engineering by using MOFs as functional coatings, localized reinforcements, or stimuli-responsive modifiers. For instance,

- MOF coatings on metals: MgCu-MOF-74 anchored on a Ti alloy using polydopamine forms a robust coating. This coating can maintain structural stability while delivering therapeutic ions, without compromising the substrate’s inherent load-bearing capacity 142.

- Rigid-soft hybrid system: A bioactive implant interface was designed by Li *et al*. that incorporated a multi-nanozyme hydrogel (BPQD@Cu-MOF) into a 3D-printed porous Ti-6Al-4V scaffold. This “rigid-soft” hybrid system has the primary load-bearing of Ti (compressive modulus ~15.3 GPa), while the MOF provides bioactivity and controlled-release capability. This showed the feasibility of weight-bearing defect repair under complex conditions like diabetic mellitus 143.

- Hierarchical Ti-6Al-4V implant with MOF-loaded hydrogels: Wang *et al*. created a hierarchical 3D-printed Ti-6Al-4V implant coated with an ECM-like silk fibroin hydrogel encapsulating drug-loaded Mg-MOF-74 nanoparticles. This construct reached an elastic modulus of ~3.4  GPa and a yield strength of ~71 MPa, making it suitable for fixing cortical bone. Additionally, the MOF component could further regulate immunomodulation and osteogenesis 60.

- MOF-reinforced bone cements: Wang *et al*. created a biodegradable bone cement by incorporating Mg-MOF into a mixed matrix of calcium sulfate/calcium citrate/DCPA. The rigid coordination structure of the MOF improved compressive strength from 27  MPa to 32  MPa through H-bonding interactions within the cement matrix, while also providing antibacterial and immunomodulatory functions 144.

In summary, repairing load-bearing bone requires strong, stable materials, such as metals, reinforced cements, and porous bioceramics. These examples showed that with coatings, hybrids, or composites, MOFs can be both bioactive and structurally sound.

## 6. Multifunctional MOF Platforms: Beyond Osteogenesis

### 6.1. Combating Bacterial Infection: Antibacterial Ion and Drug Release

Globally, there are 178 million cases of bone fractures that occur every year. Of these, about 5% progress to infections, affecting nearly 1.8 million patients 145. Infections impose a substantial economic burden, with hospital costs 4-8 times higher than for patients without infections 146. Bacterial infections, along with the resulting inflammatory cascade, severely compromise bone healing by damaging host cells and disrupting the local osteogenic microenvironment 147.

**Figure 9:**
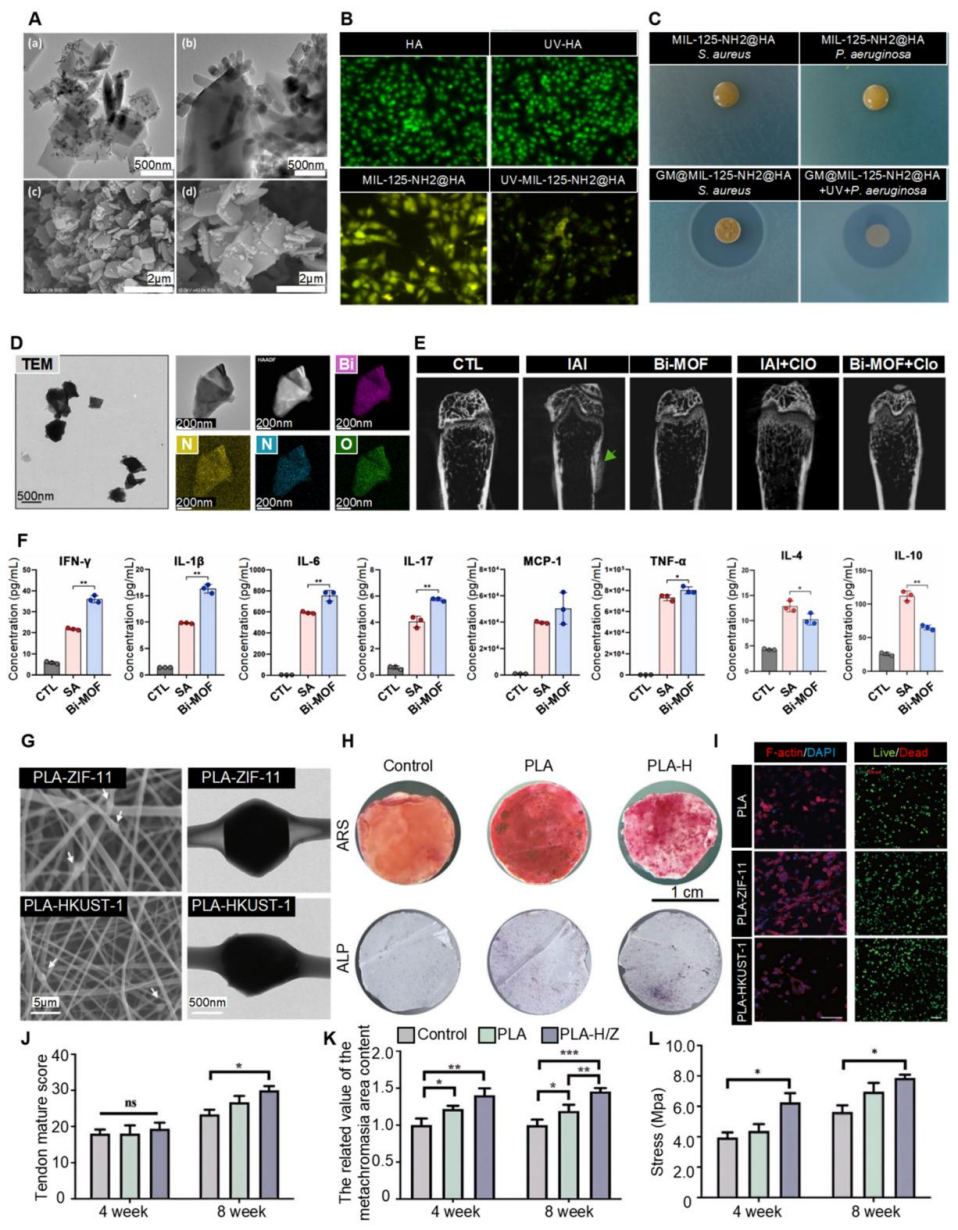
**MOF-Based Nanocomposites for Anti-Infection and Osteogenic Applications. (A)** TEM images showing structural characterization of MIL-125-NH_2_@HA. **(B)** Cell viability was assessed with live/dead staining. **(C)** Bactericidal assay against *S. aureus* and *E. coli* showing colony formation. **(D)** Compositional analysis of Bi-MOF, TEM image with corresponding elemental mapping. **(E)** *In vivo* infection model evaluation using micro-CT reconstructions of infected femoral defects. **(F)** Immune modulation analysis: Protein levels of key antibacterial cytokines secreted by infected macrophages. **(G)** Composite material morphology showing with SEM images, ZIF-11 particles indicated by arrows. **(H)** Osteogenic differentiation assessment: ALP staining (day 7) and ARS staining (day 21). **(I)** Immunofluorescence staining of the cytoskeleton combined with live/dead staining to assess cell adhesion and viability. **(J-L)** Tendon regeneration quantification: **(J)** Histological tendon maturity score, **(K)** Quantitative analysis of glycosaminoglycan content via alcian blue staining, and **(L)** Biomechanical evaluation of tensile strength in regenerated tendon tissue. Adapted with permission from 149, copyright 2023 Elsevier; 155, copyright 2021 American Chemical Society; 164, copyright 2021 Wiley.

MOFs offer a promising alternative to conventional antibacterial agents. They have optimized topologies, long-lasting release, and thermal/chemical stability, making them ideal for antimicrobial applications 148. A recent study made a special antibacterial composite by integrating HA with MIL-125-NH_2_ and loading it with gentamicin (GM) **(Figure 9A)**. This GM@MIL-125-NH_2_@HA composite exhibited excellent biocompatibility with osteoblasts and fibroblasts **(Figure 9B)**. Importantly, the MOF component acts as an effective carrier of antibiotics, allowing a rapid initial release followed by slow, sustained release of gentamicin, which proved highly effective against both *S. aureus* and *P. aeruginosa*, significantly reducing their growth** (Figure 9C)**. This dual-phase release gives immediate and long-lasting antimicrobial action, making MOF-based composites promising for preventing postoperative infections and enhancing the safety of bone implants 149.

Intrinsic bactericidal ion release from MOF nanomaterials is a popular strategy to prevent infection 150. Zhang *et al.* designed biomimetic MOF structures incorporating both Cu^2+^ and Zn^2+^. In this MOF system, Zn and Cu ions have complementary antibacterial effects, Zn^2+^ released from ZIF-8 exhibited MIC values of 125-500 μg/mL, while Cu^2+^ released from Cu-MOFs showed MICs of 64-256 μg/mL 151. These ions synergistically disrupted bacterial wall integrity, while promoting pre-osteoblast recruitment and differentiation via phytic acid-mediated biomineralization.

Beyond intrinsic ion release, MOFs can also carry antibiotics or antimicrobial peptides. Yan *et al.* prepared a multifunctional fluorine-doped Zr-MOF film on Ti implants using fumaric acid as both an organic ligand and an anti-inflammatory agent. This platform showed robust bactericidal ability against both Gram-positive and Gram-negative bacteria, and helped modulate macrophage activities toward an anti-inflammatory state 89. Zhu* et al.* created a 3D-printed scaffold composed of PCL/HAp and Cu-MOF. They found that using low MOF concentrations (0.05%-0.2%) was good for balanced antibacterial and osteogenic functions. At high MOF concentration of 1%, its inhibition rates could reach 90.07% (against *S. aureus*) and 80.03% (against *E. coli*), but at the expense of cell viability and osteogenesis of BMSCs 152.

However, several challenges need to be addressed for future clinical translation. Long-term exposure to metal ions might lead to bacterial resistance through metabolic changes like efflux pump activation or genetic adaptation. Also, the narrow therapeutic window between antibacterial efficacy and cytotoxicity still requires further careful optimization. Future bactericidal designs should modulate antibacterial activity based on the infection status, to avoid excessive inflammation as healing progresses.

### 6.2. Modulating the Immune Microenvironment

The emerging field of osteoimmunology highlights the intricate crosstalk between immune cells and skeletal tissues, with macrophage polarization playing a decisive role in bone regeneration 153. Promoting the polarization of M1-like macrophages into M2-like macrophages is crucial for establishing a favorable osteoimmune environment. M2 macrophages facilitate bone repair with the secretion of many cytokines and factors, including TGF-β, BMPs, and IL-10, which collectively enhance mesenchymal stem cell osteogenic differentiation and angiogenesis 154.

MOFs have emerged as promising immunomodulatory agents, especially in combating persistent infections. MOFs offer sophisticated strategies to modulate the immune landscape through multiple approaches. Yang *et al.* demonstrated that bismuth-based MOFs (Bi-MOFs) act as efficient intracellular hydrogen sulfide (H_2_S) scavengers **(Figure 9D)**. By inhibiting H_2_S-mediated S-sulfhydration of HIF-1α, Bi-MOFs stabilize and reprogram macrophages toward an antibacterial phenotype, enhancing innate and adaptive immunity, facilitating bacterial clearance **(Figure 9E),** and promoting long-lasting protective immunity **(Figure 9F)**. These findings underscore the significant potential of MOFs in immune modulation, offering a novel therapeutic strategy for managing stubborn infections such as those associated with medical implants 155.

In another study, Sun *et al.* developed a Kartogenin-loaded nanogel system (KZIF@HA) that improved cartilage tissue permeability by 40% compared to free KGN, promoted M2 polarization, increased IL-10 secretion, and inhibited JNK and ERK pathways in chondrocytes 156. In a complementary approach, Ge *et al*. developed Ket@Mg-MOF-74, which reduced cyclooxygenase-2 (COX-2) expression and modulated the secretion of osteogenic cytokines and pro-inflammatory factors. In this system, released Mg^2+^ may influence macrophage polarization via NF-κB and MAPK signaling pathways, though further mechanistic studies are needed 157.

### 6.3 MOF-Based Drug Delivery to Promote Angiogenesis

Successful vascularized osteogenesis requires coordinated angiogenesis and osteogenesis. Adequate vascularization supplies oxygen, nutrients, and progenitor cells, while impaired blood flow can cause bone necrosis and a compromised osteogenic microenvironment 158.

MOFs support vascularized bone formation through multiple mechanisms. Li *et al.* encapsulated deferoxamine within ZIF-8, extending its half-life and promoting extensive vascular network formation *in vivo*. This system enhanced type H vessel formation, a crucial step in coupling angiogenesis and osteogenesis, and was associated with enhanced bone regeneration, evidenced by increased osteocalcin and BMP-2 expression. Micro-CT angiography and CD31 immunohistochemistry confirmed neovascularization promoted by MOF-based systems 159.

Other strategies include MOF-mediated delivery of VEGF, platelet-derived growth factor (PDGF), and Co-MOFs that mimic hypoxia and activate endogenous angiogenic pathways by stabilizing HIF-1α. Zheng *et al.* fabricated IL-4-MOF@CaP, a pH-sensitive MOF encapsulating IL-4 as a multifunctional platform integrating immunomodulation, angiogenesis, ROS scavenging, and mineralization, through IL-4 release, providing Mg^2+^, gallic acid, and calcium/phosphate 160. Beyond delivering specific factors, MOFs can be intrinsically functionalized to promote angiogenesis. Si *et al.* demonstrated that CuO@ZnO coatings on Ti implants enhanced HUVEC angiogenesis via Cu2*+-*induced VEGF upregulation 161

### 6.4. Anti-Inflammatory and Antioxidant Effects

Elevated ROS levels disrupt bone homeostasis by suppressing osteoblast activity and promoting osteoclast formation. MOFs counter these effects through two primary mechanisms: (1) delivery of antioxidant agents, and (2) intrinsic nanozyme activity enabling catalytic ROS decomposition.

Certain MOFs exhibit intrinsic enzyme-mimetic properties that enable efficient ROS decomposition. Fe-MOFs, with peroxidase (POD)-like activity, catalyze H_2_O_2_ breakdown 162. Shu *et al.* synthesized Zn/Co-MOF-modified β-TCP with broad-spectrum ROS-scavenging capabilities, protecting BMSCs and chondrocytes from oxidative stress while promoting osteogenic differentiation and chondrocyte maturation 130. Liu *et al.* designed two cerium-based MOFs as superoxide dismutase mimics (Ce(III)-BTC and Ce(IV)-BTC) that efficiently eliminate superoxide via electron transfer 163.

### 6.5 Tendon-to-Bone Interface Regeneration

MOFs are also a highly promising platform for the complex challenge of tendon-to-bone interface regeneration. They can release bioactive metal ions steadily and in a controlled space. A bipolar, flexible membrane made from electrospun fibers used two different MOFs: ZIF-11 (a Zn-based framework) on one side and HKUST-1 (a Cu-based framework) on the other side **(Figure 9G)**. This design is similar to the natural structure of the tendon-to-bone interface. The sustained release of Zn^2+^ helps tendon cells grow, increases tendon fibroblast activity and COL-1 synthesis. Cu^2+^ release aids osteogenic differentiation, biomineralization **(Figure 9H)**, and biocompatibility **(Figure 9I)**. In a rat rotator cuff repair model, this MOF-based scaffold facilitated fibrocartilage reconstruction and restored biomechanical strength **(Figure 9J-L)**. This new strategy showed the potential of multi-functional MOF composites to orchestrate the coordinated healing of gradient tissues with high mechanical requirements 164.

In summary, MOFs have many applications beyond promoting bone healing. They also control infections, regulate immune response, support angiogenesis, and reduce oxidative stress. Combining these capabilities into a single platform represents a paradigm shift in bone tissue engineering, generating a smart orthopedic implant that can dynamically respond to and modulate the healing environment. Future studies should focus on optimizing the spatiotemporal control of these multiple functionsto more closely mimic the natural tissue-healing process.

## 7. Current Challenges and Future Perspectives

Although preclinical studies often show that MOFs are effective, their clinical translation remains uncommon. The main problems involve biosafety issues, degradation kinetics, manufacturing scalability, and the need for phase-specific therapeutic delivery. A comparative overview of the major MOF systems is crucial for identifying the issues they face in this process. Each MOF system has a unique profile of bioactive components, mechanisms, and target applications, which in turn, lead to specific limitations **(Table 1)**.

### 7.1. Biosafety and Specific Design

The clinical efficacy of MOF-based strategies relies on how well they fit into the natural bone repair process: inflammation, repair (soft callus formation), and remodeling. However, current MOF systems often deliver osteogenic factors (*e.g.*, Sr^2+^, BMP-2) too early during the inflammatory phase, potentially exacerbating inflammation and delaying repair. Future “smart” systems need to detect and respond to phase-specific signals. For example, secreting anti-inflammatory cytokines (*e.g.*, IL-4 or IL-10) in high inflammation conditions (elevated levels of TNF-α or MMP-9); releasing osteogenic factors when CRP is low, or ALP goes up; and starting remodeling using OPG when TRAP levels rise 165. Therefore, future work should focus on: (1) designing MOFs with precise degradation profiles and ion-release kinetics, by using core-shell structures, composite matrices, or stimuli-responsive linkers, to ensure release when needed and stay safe; (2) Defining the safe concentration window of metal ions that maximizes therapeutic effect while minimizing toxicity; and (3) Evaluating long-term immune compatibility, specifically assessing the impact of ion release on macrophage phenotype dynamics, cytokine secretion profiles, and the foreign body reaction over time.

### 7.2. Manufacturing Scalability and Clinical Translation

Producing MOFs in large amounts with high purity is still difficult because of costly raw materials, low synthesis efficiency, and complex downstream processing 166. Although new technologies like electrospinning and 3D printing have enabled the integration of MOFs into various biomaterials, most current optimization often relies on empirical approaches. The lack of systematic design can restrict research efficiency, consume too many resources, and hinder the manufacturing scalability and clinical translation.

Artificial intelligence (AI), machine learning, and computational modeling have recently overcome these challenges effectively. They can now accurately predict the physicochemical characteristics of material properties, help improve the compositions and synthesis parameters, and greatly accelerate progress and reduce costs 167. However, they need high-quality, comprehensive data and guidance to function properly. Future directions include: (1) Standardized safety and biocompatibility evaluation protocols, including long-term biodistribution studies, immune cell profiling, and genomic toxicity assessments specific to MOF degradation products. (2) scalable, cost-effective, and green manufacturing (continuous flow synthesis, mechanochemical preparation, or 3D printing-assisted in situ MOF growth) to facilitate clinical-grade production. (3) encouraging multidisciplinary collaborations bridging material science (patient-specific implants, mixed MOF-composites) and clinical practice 168.

Some promising approaches include coatings on Ti implants 169, addition to bone cement 170, and incorporation into hydrogels 171. These approaches use the natural mechanical properties of implants/materials while using MOFs for localized, controlled therapeutic release. MOFs with ions like Mg^2+^ 172, Zn^2+^ 173, and Ca^2+^ 141 are common because they are safe and bioactive, helping to address complex, multifactorial pathologies, such as infected or diabetic bone defects. But, overly complex “all-in-one” designs that combine many functions, *e.g.*, drug delivery, gene transfection, and imaging, within a single implant often result in compromised outcomes and unpredictable *in vivo* behavior.

### 7.3. Key Advantages and Limitations of MOF Biomaterials

MOFs overcome the static, bio-inert nature of many bioceramics. They offer dynamic functionality, deliver therapeutic agents, and promote tissue repair 30. More importantly, MOFs provide osteogenic, angiogenic, and antibacterial functions that bioceramics lack. Although polymers (membranes, fibers, hydrogels) have great biocompatibility and processability, their bioactivity often depends on the adsorption or encapsulation of exogenous factors 174. This leads to poor healing outcomes because of low loading efficiency, burst release, and degradation. Unlike polymers, MOFs combine the programmability and protection of advanced nanocarriers with the structural integrity and bioactivities 175.

Despite these advantages, MOFs face distinct challenges, and their limitations include: (1) weak mechanical strength compared to metallic implants and bioceramics, which restricts their use as a load-bearing implant, (2) unpredictable long-term degradation kinetics, raising concerns about *in vivo* fate, and (3) high cost and complexity of clinical-grade synthesis. Nevertheless, MOFs are promising and revolutionary for bone tissue engineering. Ongoing collaborations across materials science, bioengineering, and clinical medicine are important. MOF-based biomaterials are ultimately expected to pave the way not only for orthopedic use, but also in broader realms of regenerative and precision medicine.

## 8. Conclusions

In recent decades, MOF-based biomaterials have represented a paradigm-shifting platform in bone tissue engineering. Their tunable structures, high surface area, and multifunctionality enable the sustained ion release, controlled delivery of biomolecules and drugs, or the ability to integrate into advanced composite orthopedic implants. With advances in bioengineering, MOF-based biomaterials promote osteogenesis, angiogenesis, and immunomodulation while combating infection and inflammation. Future progress will depend on developing smart, stimuli-responsive systems and integrating computational design with biocompatible components. By bridging material innovation with biological principles, MOF-based strategies hold great potential to overcome current limitations in bone defect repair. However, clinical translation requires addressing key challenges in long-term biosafety, degradation kinetics, and scalable manufacturing, which should be considered in the development of MOF-based biomaterials for bone defect repair.

## Figures and Tables

**Figure 1:**
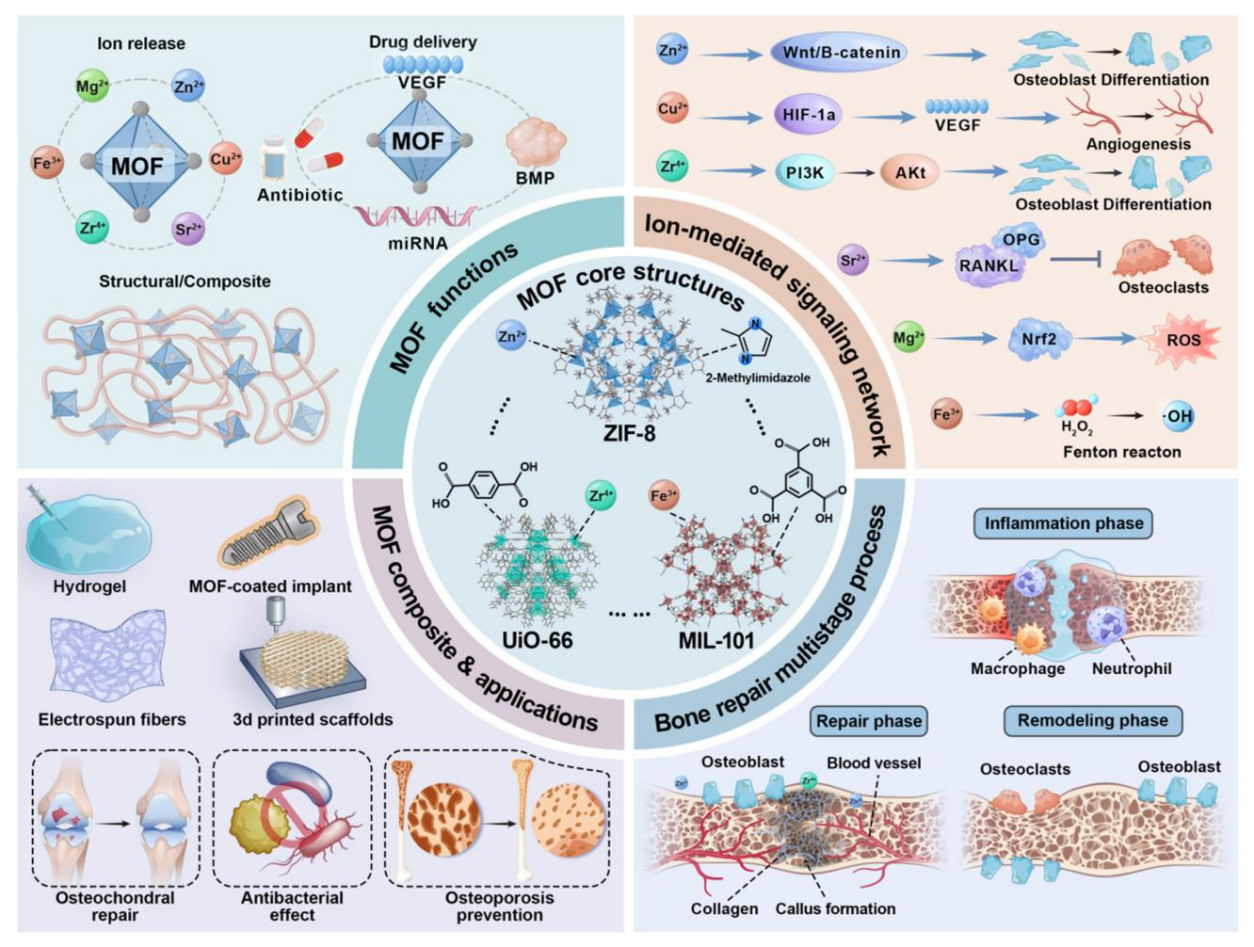
The newly developed MOF-based biomaterials have been widely used for bone defect repair. Currently, there are many different types of MOFs, such as ZIF-8, UiO-66, and MIL-101. MOFs are composed of metal nodes and organic linkers and can serve as platforms for controlled metal ion release and drug delivery due to their special structural and compositional properties. In the context of bone defect repair, MOF-based materials can be manufactured into various biomaterials to support bone healing, such as hydrogel, metallic implants, fibers, or scaffolds. At the molecular level, MOF-based biomaterials can be designed to activate specific signaling pathways, thereby modulating osteogenesis, angiogenesis, and other biological effects. With all of these merits, MOFs play an important role in regulating bone metabolism and accelerating the repair of bone defects.

**Table 1:** Comparative Analysis of Representative MOF Systems for Bone Regeneration

Category	MOF	Core	Primary Functions	Key Mechanisms	Applications	Limitations
1. Essential Ion-Based MOFs	Mg-MOFs (e.g., Mg-MOF-74)	Mg^2+^	Osteogenesis and BMSC differentiation 71-73	Sustained Mg^2+^ release activates Nrf2/MAPK pathways.	Age-related bone defects	Fast degradation kinetics
			Angiogenesis (VEGF/HIF-1α upregulation) 72	Creates an alkaline, pro-regenerative microenvironment.	Diabetic bone healing	Relatively low mechanical strength
			Immunomodulation (M2 polarization) 71		Defects requiring immunomodulation	
			Anti-senescence (ROS scavenging) 71			
	Zn-MOFs (e.g., ZIF-8)	Zn^2+^	Osteogenesis (Wnt pathway activation) 85	pH-sensitive degradation targets acidic sites (infection/inflammation).	Infected bone defects	Potential for burst release in acidic environments
			Antibacterial activity 150	Zn^2+^ acts as a signaling messenger and an enzymatic cofactor.	Inflammatory bone conditions	Long-term in vivo fate of nanoparticles
			Anti-inflammatory and antioxidant 77, 85	AKT/GSK3β/NRF2 signaling		
			pH-responsive drug delivery 77, 106	Wnt signaling		
	Sr-MOFs (e.g., Sr-doped ZIF-8)	Sr^2+^	Dual-action bone metabolism (pro-osteoblast, anti-osteoclast) 54, 81	Sr^2+^ modulates the RANKL/OPG signaling.	Osteoporotic fractures	Long-term systemic effects of Sr^2+^ accumulation require study.
			Immunomodulation (M2 polarization) 81		Conditions with excessive bone resorption	
			Effective in diabetic models 82			
2. Therapeutic Transition Metal MOFs	Cu-MOFs (e.g., L-Asp-Cu(II) MOF)	Cu^2+^	Angiogenesis-osteogenesis coupling 74	Cu^2+^ stabilizes HIF-1α, upregulating VEGF and activating TGF-β/BMP pathways.	Critical-sized bone defects	Narrow therapeutic window (cytotoxicity risk)
			Neuro-vascular-bone regeneration 75		Implant coatings for rapid osseointegration	Requires precise control over release kinetics
			Inherent antibacterial property			
	Co-MOFs (e.g., ZIF-67)	Co^2+^	Hypoxia mimicry (promotes chondrogenesis and angiogenesis) 84	Co^2+^ stabilizes HIF-1α, mimicking a hypoxic microenvironment.	OC defects	Significant biosafety concern: Potential cytotoxicity limits clinical translation.
			Antioxidant activity (ROS scavenging) 85		Ischemic bone repair (with stringent safety design)	
3. Structural and Functional MOFs	Zr-MOFs (e.g., UiO-66-NH_2_)	Zr^4+^, Functional ligands (*e.g.*, -NH_2_)	Excellent biocompatibility and osteoblast support 87, 88	An extremely stable framework enables sustained, long-term release.	Coatings for permanent implants	Very slow biodegradation (potential permanent foreign body)
			High-capacity, stable drug delivery platform 88	Surface functionalization enhances bioactivity.	Long-term local drug delivery systems	Low intrinsic bioactivity relies on the loaded cargo
			Antibacterial (with doping, *e.g.*, F-) 89			
	Fe-MOFs (e.g., MIL-100(Fe))	Fe^2+^/^3+^	Catalytic therapy (nanozyme: POD-like activity) 162, 163	Fenton/Fenton-like reactions for ROS generation/scavenging (chemodynamic therapy).	Bone defects with high oxidative stress (*e.g.*, rheumatoid arthritis)	Complex iron metabolism *in vivo*
			High drug-loading capacity 79, 149		Stimuli-responsive drug carriers	Risk of disrupting iron homeostasis
4. Smart MOF Systems	Bimetallic MOFs (e.g., Mg/Cu-MOF, Zn/Co-MOF)	Multiple ions (*e.g.*, Mg^2+^/Cu^2+^, Zn^2+^/Co^2+^)	Synergistic multifunctionality (*e.g.*, osteogenesis + angiogenesis + antibacterial) 92	Co-release of ions tailors the therapeutic milieu.	Complex, multifactorial pathologies (*e.g.*, infected diabetic defects)	Complex synthesis and characterization
			Enhanced catalysis (cascade ROS scavenging) 93	Heterometallic centers enable advanced nanozyme activities.	Interface tissue regeneration (*e.g.*, tendon-bone) 164	Risk of antagonistic effects or compounded toxicity
	Stimuli-Responsive MOFs (e.g., pH-responsive ZIF-8, NIR-responsive MAO+ZI)	Zn^2+^, Cu^2+^, Iodine	Targeted therapy in pathological microenvironments (pH, ROS) 77, 106, 108	Exploits disease hallmarks (acidity, ROS) for selective activation.	Targeted infection control (*e.g.*, biofilm eradication)	Dependency on (sometimes heterogeneous) pathological signals
			On-demand antibacterial/drug release with external triggers (light, US) 113, 115	Enables spatiotemporally precise intervention.	Tumor-associated bone defects	Requires external devices for triggering

## Data Availability

The data used and/or analyzed during the study are available from the corresponding author on reasonable request.

## Abbreviations

3D: Three-dimensional
AI: Artificial Intelligence
ALP: Alkaline Phosphatase
ARS: Alizarin Red S
β-TCP: β-Tricalcium Phosphate
Bi: Bismuth
BMSC: Bone Marrow Mesenchymal Stem Cell
BMP: Bone Morphogenetic Protein
BV/TV: Bone Volume / Total Volume
CAT: Catalase
COF: Covalent Organic Framework
COL-I: Collagen I
COX-2: Cyclooxygenase-2
CRP: C-Reactive Protein
Cu: Copper
DAPI: 4',6-Diamidino-2-Phenylindole
DEX: Dexamethasone
ECM: Extracellular Matrix
EV: Extracellular Vesicle
FDA: Food and Drug Administration
FRI: Fracture-Related Infection
GelMA: Gelatin Methacryloyl
GO: Graphene Oxide
HA: Hydroxyapatite
hADSC: Human Adipose-Derived Stem Cell
HIF-1α: Hypoxia-Inducible Factor 1α
hPDLC: Human Periodontal Ligament Cell
HUVEC: Human Umbilical Vein Endothelial Cell
IL: Interleukin
IRMOF: Isoreticular Metal-Organic Framework
LIPUS: Low-Intensity Pulsed Ultrasound
MAPK: Mitogen-Activated Protein Kinase
Mg: Magnesium
MIL: Materials of the Institute Lavoisier
miRNA: MicroRNA
MMP: Matrix Metalloproteinase
Mn: Manganese
MOF: Metal-Organic Framework
MRSA: Methicillin-Resistant Staphylococcus Aureus
MSC: Mesenchymal Stem Cell
MWCNT: Multi-Walled Carbon Nanotube
NIR: Near-Infrared
NP: Nanoparticle
OA: Osteoarthritis
OCN: Osteocalcin
OGP: Osteogenic Growth Peptide
OP: Osteoporosis
OPG: Osteoprotegerin
OPN: Osteopontin
PCL: Polycaprolactone
PCN: Porous Coordination Network
PDGF: Platelet-Derived Growth Factor
PDLSC: Periodontal Ligament Stem Cell
PEEK: Polyetheretherketone
PEG: Polyethylene Glycol
PI3K: Phosphoinositide 3-Kinase
PLGA: Poly (Lactic-co-Glycolic Acid)
PSM: Post-Synthetic Modification
RANKL: Receptor Activator of Nuclear Factor-κB Ligand
RNS: Reactive Nitrogen Species
ROS: Reactive Oxygen Species
Runx2: Runt-Related Transcription Factor 2
SDT: Sonodynamic Therapy
SEM: Scanning Electron Microscopy
siRNA: Small Interfering RNA
SOD: Superoxide Dismutase
Sr: Strontium
TEM: Transmission Electron Microscopy
TGF-β: Transforming Growth Factor-β
Ti: Titanium
TRAP: Tartrate-Resistant Acid Phosphatase
US: Ultrasound
VEGF: Vascular Endothelial Growth Factor
ZIF: Zeolitic Imidazolate Framework
Zn: Zinc
Zr: Zirconium
